# Genetic Diversity in the Interference Selection Limit

**DOI:** 10.1371/journal.pgen.1004222

**Published:** 2014-03-27

**Authors:** Benjamin H. Good, Aleksandra M. Walczak, Richard A. Neher, Michael M. Desai

**Affiliations:** 1 Departments of Organismic and Evolutionary Biology and of Physics, Harvard University, Cambridge, Massachusetts, United States of America; 2 FAS Center for Systems Biology, Harvard University, Cambridge, Massachusetts, United States of America; 3 CNRS-Laboratoire de Physique Théorique de l'École Normale Supérieure, Paris, France; 4 Max Planck Institute for Developmental Biology, Tübingen, Germany; McGill University, Canada

## Abstract

Pervasive natural selection can strongly influence observed patterns of genetic variation, but these effects remain poorly understood when multiple selected variants segregate in nearby regions of the genome. Classical population genetics fails to account for interference between linked mutations, which grows increasingly severe as the density of selected polymorphisms increases. Here, we describe a simple limit that emerges when interference is common, in which the fitness effects of individual mutations play a relatively minor role. Instead, similar to models of quantitative genetics, molecular evolution is determined by the variance in fitness within the population, defined over an effectively asexual segment of the genome (a “linkage block”). We exploit this insensitivity in a new “coarse-grained” coalescent framework, which approximates the effects of many weakly selected mutations with a smaller number of strongly selected mutations that create the same variance in fitness. This approximation generates accurate and efficient predictions for silent site variability when interference is common. However, these results suggest that there is reduced power to resolve individual selection pressures when interference is sufficiently widespread, since a broad range of parameters possess nearly identical patterns of silent site variability.

## Author Summary

A central goal of evolutionary genetics is to understand how natural selection influences DNA sequence variability. Yet while empirical studies have uncovered significant evidence for selection in many natural populations, a rigorous characterization of these selection pressures has so far been difficult to achieve. The problem is that when selection acts on linked loci, it introduces correlations along the genome that are difficult to disentangle. These “interference” effects have been extensively studied in simulation, but theory still struggles to account for interference in predicted patterns of sequence variability, which limits the quantitative conclusions that can be drawn from modern sequence data. Here, we show that in spite of this complexity, simple behavior emerges in the limit that interference is common. Patterns of molecular evolution depend on the variance in fitness within the population, and are only weakly influenced by the fitness effects of individual mutations. We leverage this “emergent simplicity” to establish a new framework for predicting genetic diversity in these populations. Our results have important practical implications for the interpretation of natural sequence variability, particularly in regions of low recombination, and suggest an inherent “resolution limit” for the quantitative inference of selection pressures from sequence polymorphism data.

## Introduction

Natural selection maintains existing function and drives adaptation, altering patterns of diversity at the genetic level. Evidence from microbial evolution experiments [1], [2] and natural populations of nematodes [3], fruit flies [4], [5], and humans [6], [7] suggests that selection is common and that it can impact diversity on genome-wide scales. Understanding these patterns is crucial, not only for studying selection itself, but also for inference of confounded factors such as demography or population structure. However, existing theory struggles to predict genetic diversity when many sites experience selection at the same time, which limits our ability to interpret variation in DNA sequence data.

Selection on individual nucleotides can be modeled very precisely, provided that the sites evolve in isolation. But as soon as they are linked together on a chromosome, selection creates correlations between nucleotides that are difficult to disentangle from each other. This gives rise to a complicated many-body problem, where even putatively neutral sites feel the effects of selection on nearby regions. Many authors neglect these correlations, or assume that they are equivalent to a reduction in the effective population size, so that individual sites evolve independently. This assumption underlies several popular methods for inferring selective pressures and demographic history directly from genetic diversity data [8]–[12]. Yet there is also extensive literature (recently reviewed in Ref. [13]) which shows how the independent sites assumption breaks down when the chromosome is densely populated with selected sites. When this occurs, the fitness effects and demographic changes inferred by these earlier methods become increasingly inaccurate [14], [15].

Linkage plays a more prominent role in models of *background selection* [16] and *genetic hitchhiking* [17], which explicitly model how strong negative and strong positive selection distort patterns of diversity at linked sites. Although initially formulated for a two-site chromosome, both can be extended to larger genomes as long as the selected sites are sufficiently rare that they can still be treated independently. Simple analytical formulae can be derived in this limit, motivating extensive efforts to distinguish signatures of background selection and hitchhiking from sequence variability in natural populations (see Ref. [18] for a recent review). However, this data has uncovered many instances where selection is neither as rare nor as strong as these simple models require [7], [19]–[24]. Instead, substantial numbers of selected polymorphisms segregate in the population at the same time, and these mutations interfere with each other as they travel towards fixation or loss. The genetic diversity in this *weak Hill-Robertson interference* [25] or *interference selection* [26] regime is poorly understood, especially in comparison to background selection or genetic hitchhiking. The qualitative behavior has been extensively studied in simulation [22], [25]–[29], and this has led to a complex picture in which both genetic drift and chance associations between linked mutations (genetic *draft*) combine to generate large fluctuations in the frequencies of selected alleles, and the occasional fixation of deleterious mutations due to Muller's ratchet. In principle, these forward simulations can also be used for inference or model comparison using approximate likelihood methods [7], [30], but in practice, performance concerns severely limit both the size of the parameter space and the properties of the data that can be analyzed in this way.

Here, we will show that in spite of the complexity observed in earlier studies, simple behaviors do emerge when interference is sufficiently common. When fitness differences are composed of many individual mutations, we obtain a type of central limit theorem, in which diversity at putatively neutral sites is determined primarily by the variance in fitness within the population over a local, *effectively asexual* segment of the genome. This limit is analogous to the situation in quantitative genetics, where the evolution of any trait depends only on the genetic variance for the trait, rather than the details of the dynamics of individual loci. We exploit this simplification to establish a coalescent framework for generating predictions under interference selection, which is based on a *coarse-grained*, effective selection strength and effective mutation rate. This leads to accurate and efficient predictions for a regime that is often implicated in empirical data, but has so far been difficult to model more rigorously. Our method also has important qualitative implications for the interpretation of sequence data in the interference selection regime, which we address in the Discussion.

## Results

### The model

We investigate the effects of widespread selection in the context of a simple and well-studied model of molecular evolution. Specifically, we consider a population of *N* haploid individuals, each of which contains a single linear chromosome that accumulates mutations at a total rate *U* and undergoes crossover recombination at a total rate *R*. We assume that the genome is sufficiently large, and epistasis is sufficiently weak, that the fitness contribution from each mutation is drawn from some distribution of fitness effects *ρ*(*s*) which remains constant over the relevant time interval. For the sake of concreteness and connection with previous literature, we will focus on the special case where all mutations confer the same deleterious fitness effect 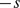, which approximates a potentially common scenario where a well-adapted population is subject to purifying selection at a large number of sites. However, our results will hold for more general distributions of fitness effects, both beneficial and deleterious, provided that individual mutations are sufficiently weak or the overall mutation rate is sufficiently large. Since the effects of linked selection are most pronounced in regions of low recombination, we devote the bulk of our analysis to the asexual limit where *R*≈0. Later, we will show that recombining genomes can be treated as an extension of this limit by means of an appropriately defined *linkage block*, within which recombination can be neglected.

These assumptions define a simple “null-model” of sequence evolution with a straightforward computational implementation (see Methods). In the present work, we focus on the genetic diversity at an unconstrained locus (e.g., a silent or synonymous site) embedded near the center of the chromosome. We focus in particular on the site frequency spectrum, 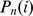, which counts the number of mutations at this locus that are shared by *i* individuals in a sample of size *n*. The pairwise diversity *π* is equal to 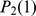 in this notation. We note that on average, 
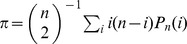
, so we can summarize the average site frequency spectrum using a combination of *π* and the relative values, 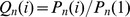. In this parameterization, *π* measures of the overall levels of diversity, while 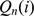 measures the shape of the site frequency spectrum. Expectations of other commonly used diversity statistics (e.g., Tajima's *D* [31] or the average minor allele frequency) can be directly computed from 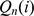.

### Background: Existing predictions break down in the interference selection regime

**Figure 1:**
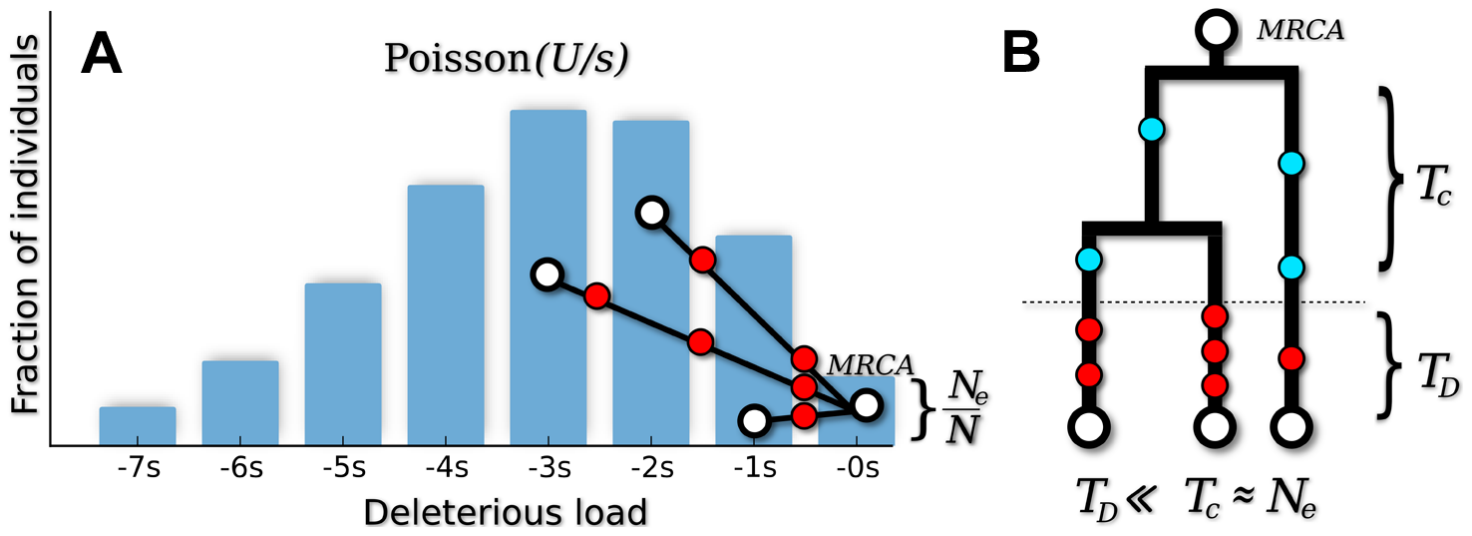
Genealogical structure in the background selection limit when 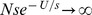. (A) In “fitness space,” the genealogy is perfectly star-like, with the most recent common ancestor (MRCA) rooted in the mutation-free class [78]. Deleterious mutations (red circles) occur every time an ancestor changes fitness classes. (B) In the standard (time-based) representation, deleterious mutations occur in a short *delay phase* of duration 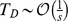, when ancestral lineages migrate through the fitness distribution. After this point, all ancestral lineages are mutation free, and coalescence proceeds according to the neutral expectation with an effective population size 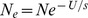. Since 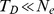, silent mutations (blue circles) will primarily occur in the coalescence phase.

Although our model is simple, the expected patterns of silent-site variability remain poorly characterized for many biologically relevant parameters. Previous theoretical work has focused on combinations of *N*, *U*, *s*, and *R* that result in relatively few selected polymorphisms per unit map length. In the limit that 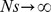, these populations converge to the *background selection limit*, where interference between deleterious mutations can be neglected and each selected site evolves independently. Traditionally, the term “background selection” is used to refer both to the general effects of purifying selection on linked neutral diversity as well as to the limiting behavior that emerges when 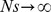. Here we use the term only in the latter sense, and we have opted for the slightly more precise label “background selection *limit*” in order to minimize confusion. This limit arises for arbitrary levels of recombination, but is easiest to visualize in the asexual case (*R*≈0). The expected fraction of individuals with *k* deleterious mutations (“fitness class *k*”) follows a Poisson distribution,
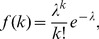
(1)where 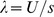 parameterizes the relative strength of mutation and selection [32]. Patterns of silent site variability are equivalent to a demographically structured *neutral* population, where the fitness classes are treated as fixed subpopulations and mutation events are recast as migration between them (see Figure 1). This is a special case of the *structured coalescent* [33], which traces the ancestry of a sample as it moves through the population fitness distribution.

The structured coalescent can be used to derive approximate analytical expressions for several simple diversity statistics [16], [34]–[38]. Previous work has shown that to lowest order in 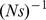, silent site diversity resembles an *unstructured* neutral population with an effective population size 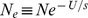. The overall level of diversity is therefore reduced from its neutral expectation (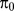) by the fraction
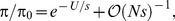
(2)while the shape of the site frequency spectrum is unchanged. Higher-order corrections, which become increasingly relevant for larger sample sizes [39], can be efficiently calculated from backward-in-time simulations of the structured coalescent (Methods) [40]–[42]. For example, in Text S1 we show that the predicted reduction in diversity is well-approximated by
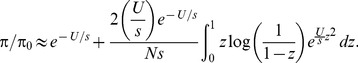
(3)provided that *Ns* is not too small.

In practice, structured coalescent methods provide reasonable accuracy for a range of parameters that we collectively term the *background selection regime*. Figure 1 shows that this constitutes a “strong-selection” region of parameter space (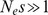), although the precise meaning of strong is somewhat different from colloquial usage. In particular, this depends on more than just the magnitude of *Ns* alone, since mutations can have selective effects that are considered strong in a single-site setting (*Ns*∼100) but nevertheless have 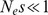 if the mutation rate is sufficiently high. Nor is this simply a statement about the magnitude of *U*/*s*. Somewhat confusingly, background selection is sometimes regarded as a “weak selection” effect, since 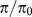 is significantly reduced only when 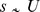. We will avoid such terminology here. Instead, we find it more productive to think of the background selection regime as a “rare interference” limit, since the distribution of fitnesses within the population coincides with the independent-sites prediction in Eq. (1).

In the present work, we focus on the opposite limit, the so-called *interference selection regime*, where mutation rates are sufficiently high or fitness effects sufficiently weak that many selected polymorphisms segregate in the population at once. In this regime, the frequencies of nearby deleterious mutations become correlated, and the distribution of fitnesses within the population fluctuates and eventually diverges from the independent-sites prediction in Eq. (1). As a result, structured coalescent methods based on this distribution start to break down (Figure S1) [36], [41], [43]. In order to predict silent site diversity in the interference selection regime, we must therefore devise an alternate method.

### Patterns of diversity “collapse” onto a single parameter family

In the interference selection regime, the twin forces of genetic drift and genetic draft generate massive deviations from the predictions described above. Yet despite the complexity of these forces, the patterns of silent-site variability display a number of striking regularities in this regime, which we now demonstrate through simulations of our evolutionary model (see Methods). This approach is similar to earlier simulation studies [22], [25]–[29], but we focus on identifying patterns that can be exploited for *prediction*, rather than simply describing the behavior observed in the presence of interference. We later generalize these patterns and use them to establish a new coalescent framework for predicting genetic diversity when interference is common.

**Figure 2:**
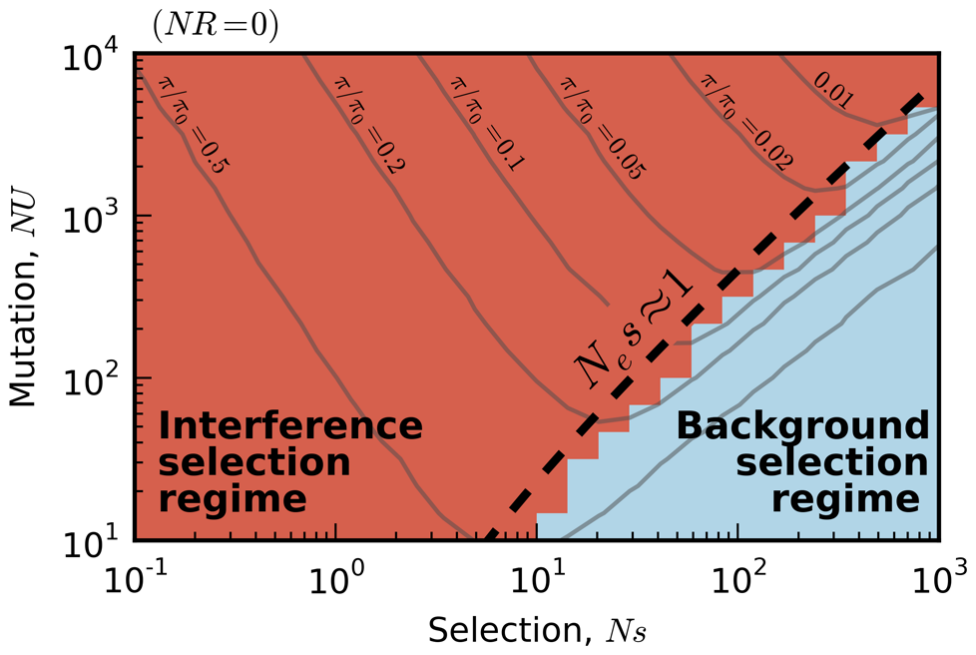
Existing predictions for silent-site diversity break down in the interference selection regime. Blue tiles denote populations where the pairwise diversity *π* falls within 50% of the background selection prediction in Eq. (2), and red tiles denote populations that deviate by more than 50%. For comparison, the solid black line depicts the set of populations with 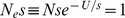, which is close to the point where Muller's ratchet begins to click more frequently [41].

**Figure 3:**
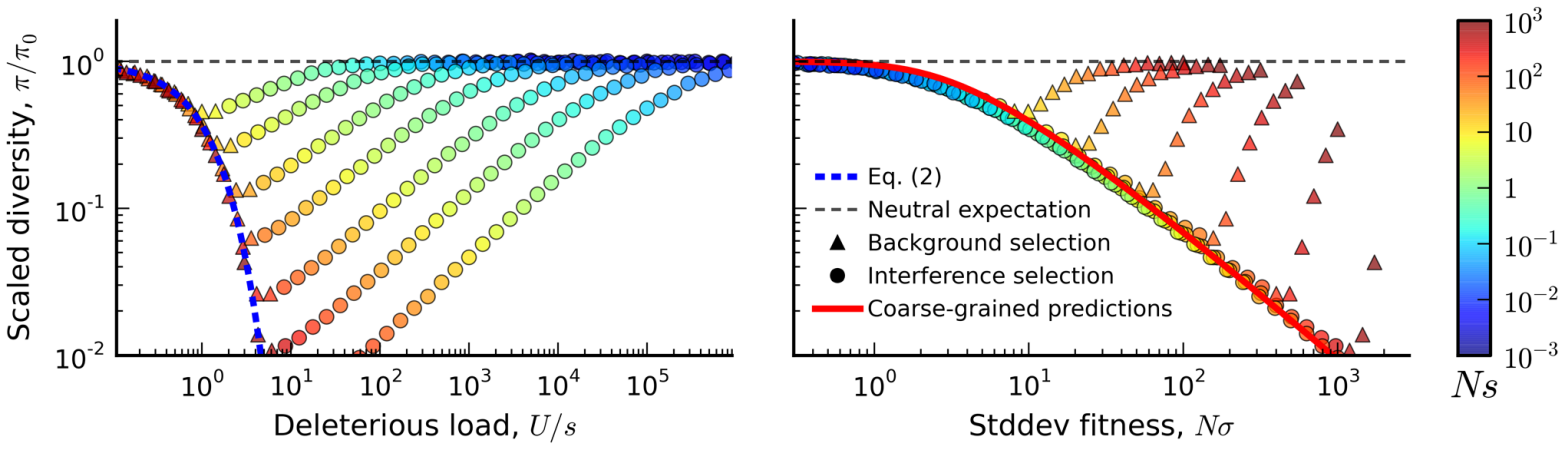
The average reduction in silent site diversity relative to the neutral expectation. Colored points are measured from forward-time simulations of the simple purifying selection scenario in Figure 2 for 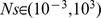 and 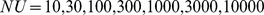. Triangles and circles distinguish populations that are classified into the “background selection” and “interference selection” regimes, respectively (see Methods). In the left panel, these results are plotted as a function of the deleterious load 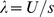, and the background selection prediction from Eq. (2) is given by the dashed line. The right panel shows the same set of results plotted as a function of the observed standard deviation in fitness, and the solid line denotes the “coarse-grained” predictions (see Methods). Note that for populations in the background selection regime (triangles), 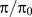 is determined primarily by the deleterious load, independent of *Ns* and *NU*. For populations in the interference selection regime (circles), 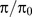 is determined primarily by the standard deviation in fitness.

First, we measured the average site frequency spectrum, 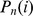, and the average fitness variance, 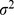, in 280 asexual populations evolving under our simple purifying selection model, where all mutations share the same deleterious fitness effect. These populations were arranged on a grid, with mutation rates (*NU*) ranging from 10 to 10^4^ and selection strengths (*Ns*) ranging from 10^−3^ to 10^3^. We distinguish between populations that fall in the background selection regime or the interference selection regime, which loosely coincide with the red and blue regions in Figure 2 (see Methods). Figure 3 shows the observed reduction in diversity, as measured by the pairwise heterozygosity π relative to its neutral expectation, 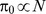. As expected, the reduction in diversity is well-approximated by Eq. (2) in the background selection regime (triangle symbols) [27], but it breaks down for populations in the interference selection regime (circles) [37]. In addition, the traditional measure of the deleterious load 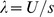 ceases to be a good predictor of diversity in the interference selection regime, with more than an order of magnitude variation in 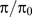 for the same value of *λ*. However, when the same populations are reorganized according to their variance in fitness (Figure 2 B), the pattern essentially flips. The variance in fitness within the population is a strikingly accurate predictor for 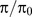 in the interference selection regime (circles), but it is a poor predictor in the background selection regime (triangles).

**Figure 4:**
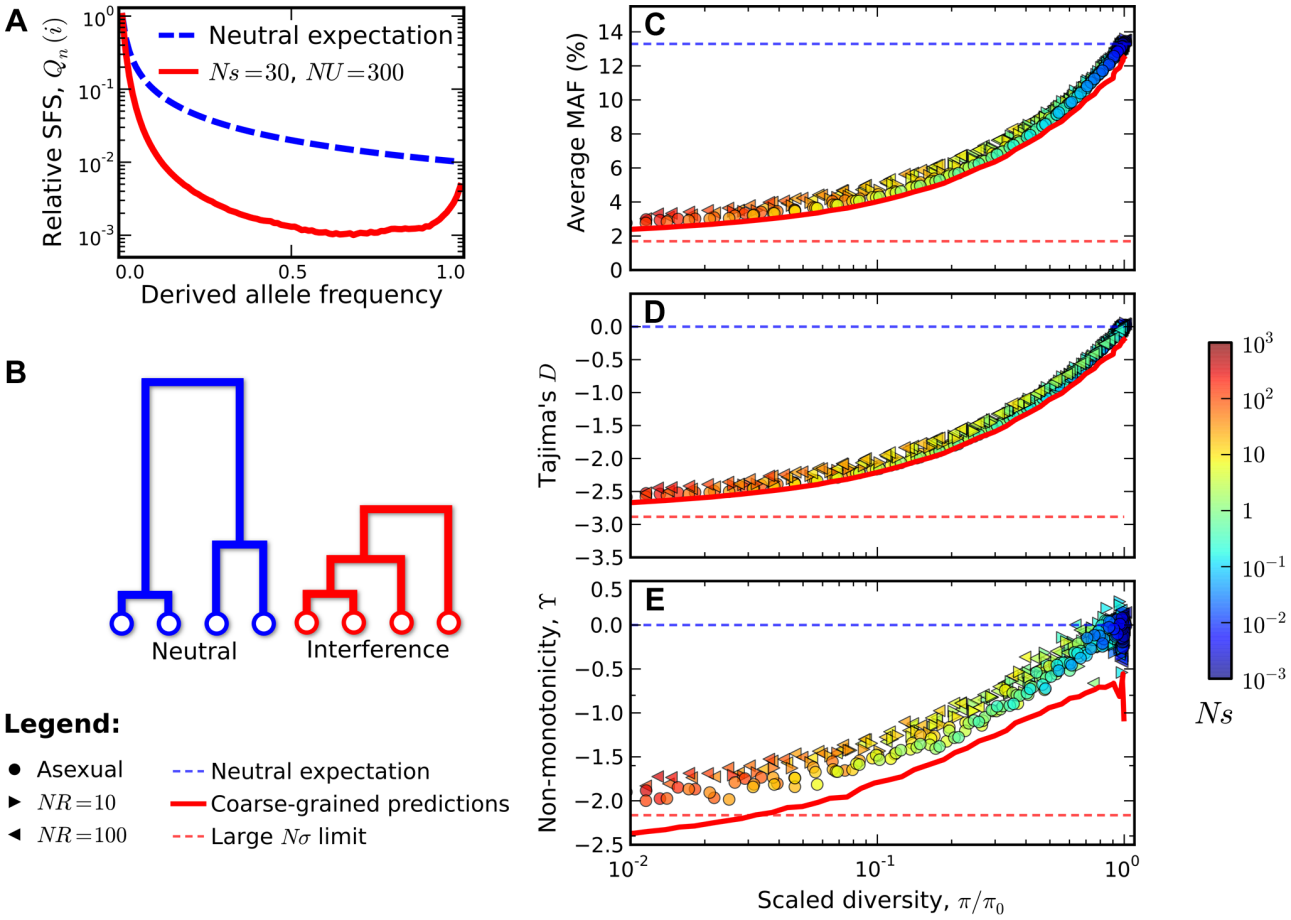
Signatures of pervasive interference selection in the silent site frequency spectrum for a sample of *n* = 100 individuals. (A) A typical example of the average site frequency spectrum in the interference selection regime, simulated for *Ns* = 30, *NU* = 300, and *R*≈0 (red line). For comparison, the neutral expectation is given by the dashed blue line. (B) A schematic illustration of the genealogical structure observed in neutral populations (left) and those subject to widespread interference (right). (C) An excess of rare alleles measured by the average minor allele frequency, (D) Tajima's *D*, and (E) non-monotonic or “U-shaped” behavior at high frequencies measured by 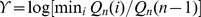. The statistics in (C–E) are plotted as a function of the reduction in pairwise diversity, 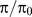. Circles denote the subset of simulations in Figure 3 that were classified into the interference selection regime, while the right- and left-pointing triangles depict an analogous set of simulations for recombining genomes with *NR* = 10 and *NR* = 100, respectively. All points are colored according to the same scale as Figure 2. For comparison, the solid red lines show the “coarse-grained” predictions (see Methods), while the dashed lines show the corresponding predictions under neutrality (blue) and for the large *Nσ* limit in Ref. [44] (red).

The distortions in the site frequency spectrum are illustrated in Figure 4. The top left panel depicts a typical site frequency spectrum in the interference selection regime, using parameters consistent with the fourth (dot) chromosome of *Drosophila melanogaster* (see Methods). Apart from an overall reduction in polymorphism, the most prominent features of this frequency spectrum include an excess of rare alleles [22], [29], and a non-monotonic (or “U-shaped”) dependence at high frequencies [44]. Since we only include silent mutations in Figure 4, the distortions in the site frequency spectrum are entirely determined by distortions in the genealogy of the sample (Figure 4 B). The excess of rare alleles is due to an increase in the relative length of recent branches, compared to more ancient ones, and the non-monotonic behavior arises from imbalance in the branching structure of the tree [22].

In the right three panels of Figure 4, we show how these distortions vary over the broad range of parameters depicted in Figure 3. For clarity, we only include populations in the interference selection regime, and we focus on the two particular features of the site frequency spectrum discussed above (the full site frequency spectra for all of the populations in Figure 3 are shown in Figure S2). Figures 2C and 2D show the excess of rare alleles as measured by the reduction in average minor allele frequency and Tajima's *D* respectively. These distortions cannot be explained by *any* constant 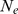, including the background selection limit. Similarly, Figure 4 E shows a measure of the non-monotonic or “U-shaped” dependence at high frequencies, using the statistic 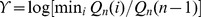. In this case, deviations from neutrality (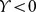) reflect topological properties of the genealogy, which cannot be explained even by a *time-dependent* 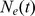. Ref. [45] showed that a “U-shaped” frequency spectrum cannot arise in *any* exchangeable coalescent model [e.g., [37], [46], [47]] unless it also allows for multiple mergers. Together, the simulations in Figure 4 show that even simple models of purifying selection can generate strong distortions in the silent site frequency spectrum, and that these distortions can persist even when individual mutations are only weakly deleterious (*Ns*∼1).

Yet the most striking feature of these distortions is not simply that they exist, but rather that they are extremely well-predicted by the reduction in pairwise diversity in these populations — which is itself well-predicted by the variance in fitness. This strong correlation is a nontrivial feature of interference selection, and it disappears for the populations that were classified into the background selection regime (Figure S3). Figure 4 also shows that correlations persist when we repeat our simulations with nonzero rates of recombination. As long as there is a sufficient density of selected mutations per unit map length, recombination seems to modify only the *degree* of the distortions from neutrality, while the qualitative nature of the distortions remains the same.

Together, Figures 3 and 4 suggest an approximate “collapse” or reduction in dimensionality from our original four-parameter model to a single-parameter curve. The evidence so far is merely suggestive, so we will revisit the generality of this result in the following sections. Yet if such a collapse exists, it carries a number of practical benefits for predicting genetic diversity in the interference selection regime: if we can predict 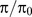, we can in principle predict *all* of the relevant patterns of silent site variability (e.g., the site frequency spectrum) even when these quantities significantly deviate from the neutral expectation. We will exploit this idea to our advantage below. However, this increased predictive capacity places fundamental limits on our ability to resolve individual selection pressures from patterns of silent site variability, even in this highly idealized setting. Our simulations suggest that in the interference selection regime, two asexual populations with the same variance in fitness will display nearly identical patterns of silent site variability, regardless of the fitness effects of the nonsynonymous mutations.

### The infinitesimal limit

The patterns that emerge from the simulations in Figures 3 and 4 reflect a fundamental limit of our evolutionary model, similar to the familiar background selection limit. To demonstrate this, we restrict our attention to nonrecombining genomes (*R* = 0), which leads to a key simplification: different genotypes with the same fitness are completely equivalent, both in terms of their reproductive capacity and their potential for future mutations. The evolutionary dynamics are completely determined by the proportion, *f*(*X*), of individuals in each *fitness class X*. The frequency of a mutant allele at some particular site can be modeled in a similar way, by partitioning *f*(*X*) into the contributions 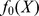 and 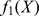 from the ancestral and derived alleles. These fitness classes evolve according to the Langevin dynamics
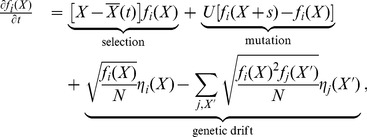
(4)where 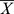 is the mean fitness of the population and 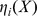 is a Brownian noise term [48]–[52]. Equation (4) decomposes the change in the frequency of the derived allele into the deterministic action of selection and mutation, and the random effects of genetic drift. It represents a natural extension of the standard diffusion limit for genomes with a large number of selected sites. Crucially, Eq. (4) tracks only the *fitnesses* of the mutant offspring as they accumulate additional mutations.

The advantage of this description is that it can be analyzed with standard perturbative techniques. For example, while the background selection limit is not always motivated in this fashion, Eq. (2) arises as a formal limit of the dynamics in Eq. (4) when 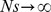 (Text S1). To avoid the trivial behavior 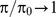, where selection can be entirely neglected, we must also take 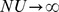 so that the deleterious load λ (and therefore 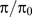) remains constant. In this limit, molecular evolution is completely determined by *λ*, or equivalently by 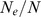, which represents the fraction of mutation-free individuals in the population. The collapse observed in the left panel of Figure 3 indicates that populations quickly *converge* to this limit when 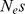 is large but finite.

Inverting this line of reasoning, a similar collapse in the right panel of Figure 3 suggests convergence to a second, *infinitesimal limit* when 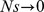. Of course, if *Ns* vanishes on its own we simply recover the neutral result, 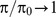. To maintain nontrivial behavior, Figure 2 B shows that we must take 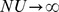 as well, so that the variance in fitness (and therefore 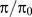) remains constant. In this way, the infinitesimal limit resembles a linked version of the infinitesimal trait models from quantitative genetics, where phenotypic variation (in this case, for the fitness “trait”) arises from a large number of small-effect alleles.

The evidence from Figure 3B is merely suggestive, but we can establish the infinitesimal limit more rigorously using Eq. (4), where it corresponds to the limit that 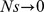 and 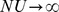 with the product 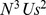 held constant. In Text S2 we demonstrate this by rescaling the moment generating function for Eq. (4); it can also be shown term-by-term using the perturbation expansion from Ref. [52]. This latter approach provides some intuition for the origin of the control parameter 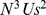. Specifically, in a nearly neutral population (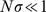), the variance in fitness is equal to
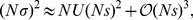
(5)which is the average mutational spread that accumulates during the coalescent timescale 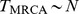. The only way that this quantity can remain finite as 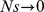 is if the product 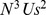 is held fixed. This argument also shows that extension of the infinitesimal limit to a distribution of fitness effects is straightforward, provided that we replace 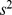 with 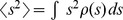. In this infinitesimal limit, the distribution of fitnesses within the population and the patterns of molecular evolution depend only on the product 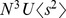 and not any other properties of *ρ*(*s*). The effects of beneficial and deleterious mutations are symmetric [44], so our analysis also applies to the long-term balance between beneficial and deleterious substitutions in finite genomes [53].

In the infinitesimal limit, selected mutations are negligible on their own, and are virtually indistinguishable from neutral mutations, but the population as a whole is far from neutral. Rather, infinitesimal mutations arise so frequently that the population maintains substantial variation in fitness, and this leads to correspondingly large distortions at the sequence level. The distribution of fitnesses within these populations is well-characterized by “traveling wave” models of fitness evolution [49], [54]–[57], which provide explicit formulae for the variance in fitness (*Nσ*) as a function of the control parameter 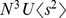 (Text S2). These formulae show that *Nσ* increases monotonically with 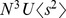, so either quantity can be used to index populations in the infinitesimal limit. We will use *Nσ* for the remainder of the paper in order to maintain consistency with Figure 3. Note that because of the pervasive interference between selected mutations, *σ* ^2^ is typically much smaller than the deterministic prediction from Eq. (1), 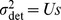, and for large *Nσ* it grows less than linearly with the number of loci under selection.

Unfortunately, patterns of molecular evolution are less well-characterized in this limit, which makes it difficult to *predict* the correlations observed in Figures 3 and 4. A complete description has been obtained only in the special cases where 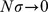 or 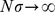. The former corresponds to a neutral population, with small corrections calculated in Ref. [52]. The latter case was recently analyzed in Ref. [44], which showed that the genealogy of the population approaches that of the Bolthausen-Sznitmann coalescent [58]. In this 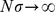 limit, silent site diversity decays as 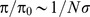, while the shape of the site-frequency spectrum, 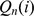, becomes independent of *all* underlying parameters. However, Figure 4 shows that many biologically relevant parameters fall somewhat far from these extreme limits, so we require an alternate method to predict genetic diversity for the moderate values of *Nσ* that are likely to arise in practice.

### Predicting genetic diversity by coarse-graining fitness

In the absence of an exact solution of the infinitesimal limit, we employ an alternate strategy inspired by the simulations in Figures 3 and 4. Convergence to the infinitesimal limit is *extremely* rapid in these figures — so rapid that we can effectively neglect any corrections to this limit all the way up to the boundary of the background selection regime. In other words, the structured coalescent and the infinitesimal limit are *both* approximately valid along this boundary. Thus, instead of using the infinitesimal limit to approximate a population with a given *Nσ*, this rapid convergence suggests that we could also use a population on the *boundary* of the background selection regime with the same *Nσ*. Intuitively, this resembles a “coarse-graining” of the fitness distribution, since it approximates several weakly selected mutations in the original population with a smaller number of strongly selected mutations in the background selection regime. On a formal level, this is nothing but a *patching method* [59] that connects the asymptotic behavior in the infinitesimal (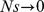) and background selection (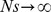) limits.

This intuition suggests a simple algorithm for predicting genetic diversity in the interference selection regime: (i) calculate *Nσ* as a function of *Ns* and *NU* as described in Text S2, (ii) find a corresponding point on the boundary of the background selection regime with the same *Nσ*, and (iii) evaluate the structured coalescent at this corresponding point. Step (ii) requires a precise definition of the boundary between the interference and background selection regimes, which we have not yet specified. Like many patching methods, this boundary is somewhat arbitrary, since the transition between the interference and background selection regimes is not infinitely sharp. Previous studies have identified several candidates (see Text S3), but in general this definition must balance two competing goals. The boundary should be close enough to the background selection limit to minimize errors in the structured coalescent. But at the same time, it must be close enough to the infinitesimal limit so that the populations rapidly converge.

**Figure 5:**
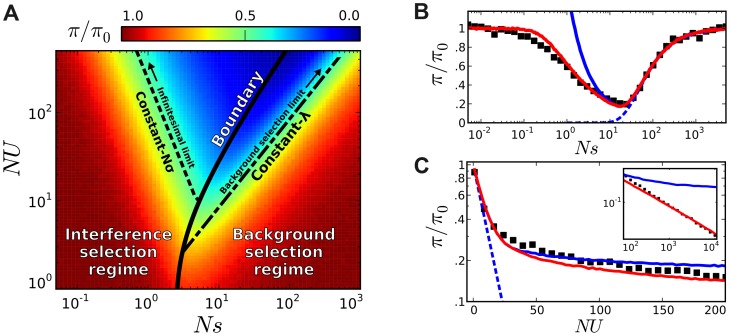
Coarse-grained predictions for the reduction in pairwise diversity. (A) The solid black line denotes the boundary separating the interference and background selection regimes, while the dashed lines to the left and right denote lines of constant *Nσ* and lines of constant *λ*, respectively. (B) A “slice” of this phase plot for constant *NU* = 50. The black squares denote the results of forward-time simulations and our coarse-grained predictions are shown in solid red. For comparison, the original structured coalescent is shown in solid blue, while the dashed line gives the prediction from the background selection limit in Eq. (2). (C) A similar “slice” of this phase plot for constant *Ns* = 10, with inset extended on a log-log scale. As 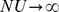, we approach the asymptotic limit 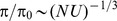 from Ref. [44].

Our definition here is based on a specific feature of the structured coalescent, which is already evident from the first-order correction in Eq. (3). For each *Nσ*, the structured coalescent starts to break down near the point of *maximum* reduction in 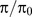, which is also close to the crossover point where Muller's ratchet starts to click more frequently [41], [50]. Together, these maxima define a “critical line” in the 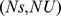 plane (Figure 5 A), which serves as the boundary between the interference and background selection regimes. Populations above or to the left of this line are classified into the interference selection regime, and the silent site variability in these populations can be predicted from the coarse-graining algorithm above. The remaining populations belong to the background selection regime, where the structured coalescent is already valid.

**Figure 6:**
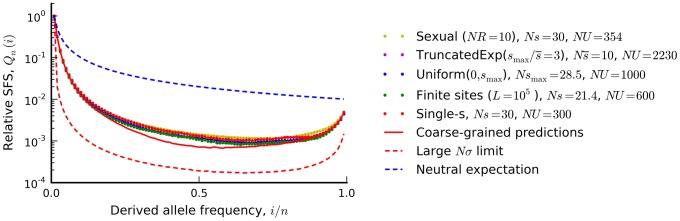
The silent site frequency spectrum from Figure 4 (red dots) and forward-time simulations of three equivalent populations predicted from our coarse-grained theory. a recombining population (yellow), a finite chromosome with *L* = 10^5^ sites that allows for beneficial as well as deleterious mutations (green), a population with a uniform distribution of deleterious fitness effects (blue), and a population with an exponential distribution of deleterious effects, truncated at 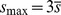. Our coarse-grained predictions are shown in solid red. For comparison, the dashed blue lines show the neutral expectation, while the dashed red lines show the large *Nσ* limit from Ref. [44] (*Nσ*≈90 in the examples above). To enable better visual comparison, each frequency spectrum is normalized by the number of singletons it contains.

We have implemented this coarse-graining procedure in a freely available Python library (see Methods), which rapidly generates predictions for the site frequency spectrum for arbitrary combinations of *Ns* and *NU*, and implements the linkage block approximation for recombining genomes described below. Other common diversity statistics (e.g., MAF or Tajima's *D*) can be computed from this site frequency spectrum as desired. Concrete examples of these predictions for the reduction in pairwise diversity are shown in Figure 5. We see that the coarse-grained predictions accurately recover the transition to the neutral limit when 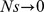 (Figure 5 B), and they also reproduce the power-law decay in heterozygosity when 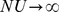 (Figure 5 C). We note that similar predictions in Figure 4 C–E (red lines) reproduce the observed distortions in the frequency spectrum statistics, while Figure 6 illustrates the predictions for the full shape of the frequency spectrum for the specific parameter combination in Figure 4 A. As is apparent from the figures, there is a broad range of parameters where the coarse-grained predictions are significantly more accurate than either the neutral expectation or the 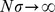 limit studied in Ref. [44].

### Distributions of fitness effects

**Figure 7:**
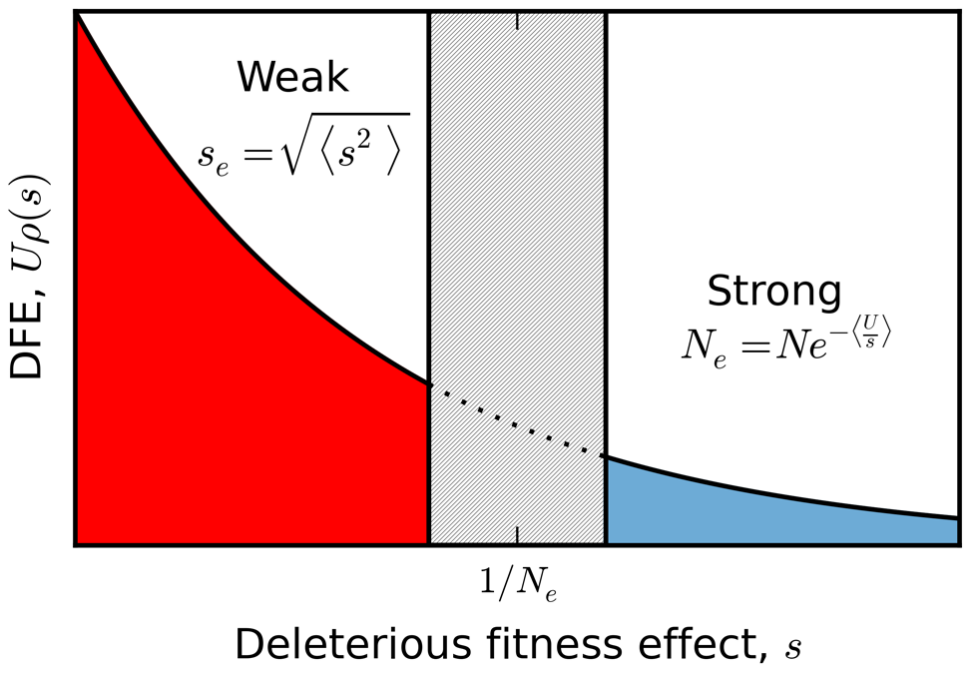
A schematic partition of a broad distribution of fitness effects. Sufficiently weakly selected mutations are described by the infinitesimal limit analyzed here, with an effective selection coefficient given by the mean squared fitness effect. Those with sufficiently strong selection coefficients generate a reduction in the effective population size according to the harmonic mean. The boundaries between these two regimes (and the width of the intermediate zone separating them) are determined self consistently by the emergent genealogical process, and vary as a function of the underlying parameters.

In order to illustrate the transition between the interference and background selection regimes, we have focused on the simplest case where all selected mutations confer the same deleterious fitness effect. However, many of our results extend to more realistic scenarios where mutations are drawn from a distribution of fitness effects (DFE). In this case, it is useful to partition the fitness effects into a weakly selected category (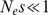) and a strongly selected category (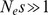), with an intermediate zone separating these two regimes (Figure 7). If the DFE is entirely contained in the weakly selected region, then our previous analysis can be easily extended. Recall that the infinitesimal limit exists for arbitrary DFEs, provided that we replace *s* with the root mean squared effect 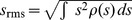 in each of the expressions above. In other words, the patterns of diversity in the infinitesimal limit are equivalent to a single-*s* DFE with an effective selection coefficient 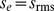. We can therefore obtain predictions for arbitrary 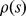 by computing 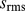 and applying our coarse-graining procedure to this corresponding single-*s* population, and we expect similar accuracy as long as the original population is sufficiently close to the infinitesimal limit. As an example, we use this procedure in Figure 6 to calculate the shape of the site frequency spectrum for a few representative DFEs consistent with the *Drosophila* dot chromosome parameters in Figure 4 A. We plot overall levels of diversity for a broader range of parameters in Figure S4. These figures illustrate the accuracy of our coarse-graining method for several different DFE shapes.

While this single-*s* mapping applies when all the mutations are sufficiently weak, there are other possible scenarios where a single effective selection strength is clearly inappropriate. For example, deleterious mutations in natural populations often span several orders of magnitude [60], which could lead to scenarios where the DFE contains a *mixture* of weakly and strongly selected mutations. A full treatment of this case is beyond the scope of the present paper, but we can illustrate the basic features with the help of a simple example. Suppose that the DFE contains two deleterious fitness effects: (i) a weakly deleterious mutation 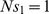 which occurs at rate 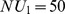 and (ii) a strongly deleterious mutation 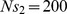 which occurs at rate 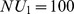. Taken individually, these mutations belong to the interference and background selection regimes, respectively. Yet the combined DFE does not belong to either regime, since it is fundamentally a mixture of the two. On the one hand, this population must fall outside of the background selection regime because the two-effect generalization of the structured coalescent [41], [61] breaks down (Figure S5). At the same time, this population cannot belong to the interference selection regime because the patterns of diversity differ from a more weakly selected population (e.g., 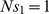, 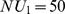, 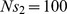, 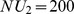) with similar variance in fitness (Figure S5).

Nevertheless, our coarse-graining procedure provides a way out of this impasse by transforming the weakly selected mutations into a form that can be handled by the structured coalescent. In this case, we note that the strongly selected mutations primarily influence the weakly selected mutations through a reduction in the effective population size, 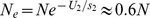. At this smaller population size, the weakly selected mutations generate a smaller variance in fitness than they would in the absence of the strongly selected mutations. Given this smaller fitness variance, we can use our single-*s* coarse graining procedure above to map the weakly selected mutations to a population on the critical line (as defined in the single-*s* case) with effective parameters 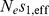 and 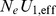. Then we can predict the patterns of diversity using the two-effect generalization of the structured coalescent, where the two effects are the strongly deleterious mutation, 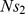, and the coarse-grained weakly deleterious mutation, 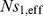 (Figure S5).

Of course, this simple two-effect example is almost as artificial as the single-*s* limit above. Ideally, we would like to generate predictions for arbitrary distributions of fitness effects. In general, we expect the qualitative behavior of these distributions to resemble the two-effect model above. Imagine for example that the DFE contains several weakly selected deleterious fitness effects and a single strongly selected effect. In this case, the weakly selected mutations can be combined into a single root-mean-square effect, 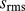, and the two-effect example above then applies. If on the other hand there are several strongly selected effects, we can account for them using a higher-dimensional structured coalescent. However, in the most general case where there is a *continuous* distribution of fitness effects, some additional complications arise. In this case, weakly selected mutations can still be coarse-grained to the infinitesimal limit, while those mutations that are sufficiently far into the strong selection regime (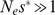) influence the evolutionary dynamics primarily through a reduction in the effective population size, 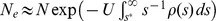. For the weakly selected mutations, this will tend to produce a smaller fitness variance and therefore a smaller deviation from neutrality than one would expect in the absence of the strongly selected mutations. However, a smaller 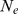 also pushes more of the strongly selected mutations into the weak selection regime, which will tend to increase the fitness variance and the corresponding deviations from neutrality. Due to these competing factors, the division between “weak” and “strong” mutations will strongly depend on the population size, the mutation rate, and the precise shape of the DFE. In addition, there may also be mutations in the intermediate region that are too strong for the infinitesimal limit to apply, but still weak enough to bias allele frequencies. For a discrete DFE, the effects of these mutations can be predicted using the structured coalescent in the appropriate number of dimensions. However, no analogous structured coalescent framework presently exists for a continuous DFE. This remains an important avenue for future work.

We note that our discussion has also ignored the effects of strongly *beneficial* mutations, which have been analyzed in several related studies [51], [62]–[66]. Unlike in the strongly deleterious case, where larger fitness effects have a smaller influence on diversity, strongly beneficial mutations tend to dominate the evolutionary dynamics if they are sufficiently common [51], [62], [64]. In this case, larger population sizes generate increased fitness variation with larger number of selected polymorphisms, and the patterns of silent site variability rapidly approach those attained in 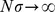 version of the infinitesimal limit [65], [66].

### Emergence of linkage blocks in recombining genomes

So far, our analysis has focused on nonrecombining genomes, but our simulations in Figure 4 show that similar behavior arises when *R*>0 as well. A formal analysis is more difficult in this case, since recombination requires explicit haplotype information and cannot be recast in terms of the evolution of fitness alone. Thus, while the structured coalescent has been extended to recombining genomes [42], [61], and an analogous version of Eq. (2) has been derived [34], [35],
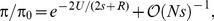
(6)there is no simple analogue of Eq. (4) that we can use to *formally* extend the infinitesimal limit.

Nevertheless, we can gain considerable insight with a simple heuristic argument, which leverages our previous analysis in nonrecombining genomes. Neighboring regions of a linear chromosome recombine much less than the genome as a whole. Sites separated by a map length 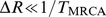 will typically not recombine at all in the history of the sample, so the ancestral process should predominantly resemble an asexual population on these length scales. On the opposite extreme, sites with 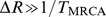 will recombine many times in the history of the sample, and will effectively act as if they were unlinked [67]. To the extent that this transition is sharp, the evolution of a recombining genome can be viewed as a collection of independent, freely recombining *linkage blocks*, each of which evolves asexually. This simple heuristic has a long history in the population genetics literature [68], [69], and it underlies many of the “sliding window” techniques used to analyze polymorphism in long genomes [70].

If each block comprises a fraction 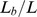 of the genome, then the distribution of fitness and the patterns of molecular evolution within each block are by definition the same as an asexual population with an effective mutation rate
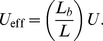
(7)Strictly speaking, the unlinked blocks also contribute to a reduction in the effective population size [46], [67], [71], [72], but we follow Ref. [73] and neglect these effects here. Given the weak population size dependence in the interference selection regime, this is often a good approximation in practice. But in principle, the logarithmic corrections from unlinked blocks can become important in extremely large genomes with a large proportion of selected sites (see Text S4 or Ref. [73] for additional discussion).

The block size itself must satisfy the condition that there are few recombination events within a block in a typical coalescence time, or
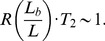
(8)Here, 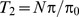 is the pairwise coalescence time for the linkage block, which is itself a function of 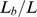 and can be calculated from Eq. (7) and the asexual methods above. Together, Eqs. (7) and (8) uniquely determine the block size in a given population. In practice, we use a generalized version of Eq. (8), 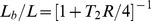, which accounts for constant factors and the saturation of the block size when 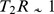. Using our coarse-grained predictions for 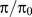, we can solve for 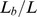 and obtain explicit predictions for the molecular evolution in recombining genomes (see Methods).

Ref. [73] has recently employed a similar argument to analyze an infinitesimal model analogous to the one studied here. They initially treat the maintenance of phenotypic (i.e., fitness) diversity as a “black box,” utilizing a top-down approach to calculate the decay of linked fitness variation caused by successive recombination events. Based on this analysis, they obtain predictions for the genetic diversity in the limit that the number of selected loci per block and the fitness variance per block become large, which, for an infinitely long genome, requires that 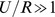 (Text S4). For recombining genomes, this plays the role of the asexual 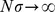 limit analyzed in Ref. [44]. Similar to the asexual case, our present analysis extends the asymptotic results of Ref. [73] to more moderate parameter values where 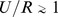. Evidence from fine-scale recombination maps [74] suggests that these parameters may be relevant for regions of reduced recombination in the autosomes of obligate sexual organisms (e.g., in humans, see Figure S6), in addition to nonrecombining sex chromosomes [29], [30] and highly selfing species such as *C. elegans* [75] where linked selection is already thought to play a large role.

**Figure 8:**
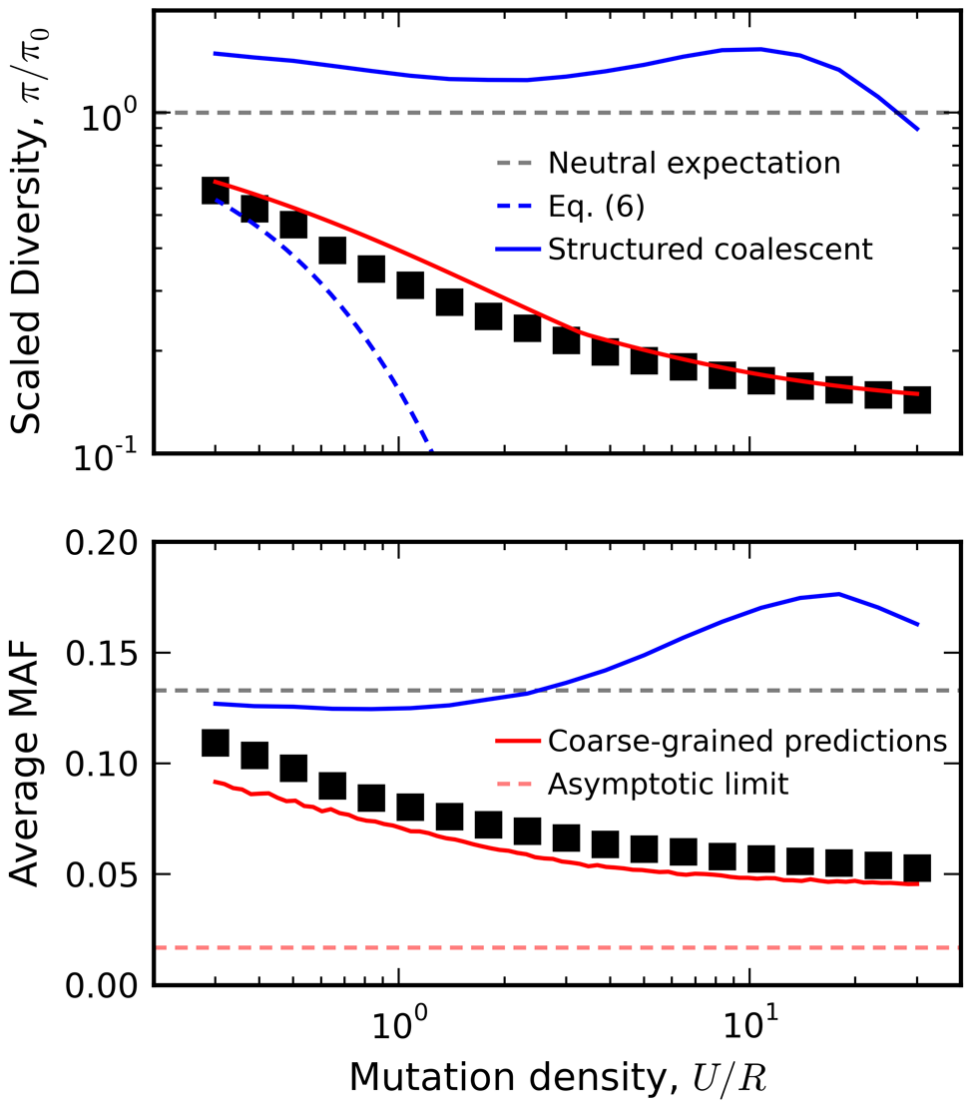
Relation between diversity and recombination rate in the presence of interference. Black squares denote the results of forward time simulations for fixed *Ns* = 10 and *NU* = 300, with recombination rates varied from *NR* = 10 to *NR* = 10^3^. Our coarse-grained predictions are shown in solid red. For comparison, we have also included predictions from the background selection limit in Eq. (6) (blue dashes) as well as the recombinant structured coalescent predictions from Ref. [42] (solid blue) and the asymptotic limit from Ref. [73] (red dashes).

As an example, we utilize this linkage block approximation to calculate the relationship between diversity and local recombination rate in Figure 8 (predictions for other quantities, e.g. the rate of Muller's ratchet, are discussed in Text S4). The reduction in minor allele frequency in particular provides a clear signature of natural selection that can be observed in human autosomal DNA (Figure S6) [7]. Interference clearly plays a large role for the populations in Figure 8, since the observed genetic diversity significantly deviates from the recombining structured coalescent [42] and the background selection limit in Eq. (2). In contrast, the crude approximation above is surprisingly accurate for these populations, even when *U*/*R* is of order one. This accuracy is especially surprising given that the predictions are obtained from an *asexual* population with a coarse-grained selection strength and mutation rate. Evidently, interference on a linear chromosome more closely resembles an asexual genome (with an appropriately defined length) rather than the freely recombining, single-site models that are more commonly employed. A more thorough investigation of the linkage block concept and its implications for other aspects of sequence diversity (e.g., linkage disequilibria, variation in recombination rate, etc.) remain an important avenue for future work.

## Discussion

Interfering mutations display complex dynamics that have been difficult to model with traditional methods. Here, we have shown that simple behavior emerges in the limit of widespread interference. When fitness variation is composed of many individual mutations, the magnitudes and signs of their fitness effects are relatively unimportant. Instead, molecular evolution is controlled by the variance in fitness within the population over some effectively asexual segment of the genome. This implies a corresponding *symmetry*, in which many weakly selected mutations combine to mimic the effects of a few strongly deleterious mutations with the same variance in fitness. We have exploited this symmetry in our “coarse-grained” coalescent framework, which generates efficient predictions across a much broader range of selection pressures than was previously possible.

Our results are consistent with previous studies that have investigated interference selection *in silico* [22], [25]–[29], [44], but our coarse-grained model offers a different perspective on the relevant processes that contribute to molecular evolution in this regime. By using the term *interference selection*, we have tried to emphasize that interference (i.e., correlations in the frequencies of selected alleles) is the distinguishing feature that separates these populations from the traditional background selection regime. Previous work, on the other hand, has argued that virtually all of the deviations from the background selection limit can be attributed to fluctuations in the fitness distribution and the effects of Muller's ratchet [22], [41], [43]. Yet our coarse-grained framework includes neither of these complications directly, and the quantitative behavior is unchanged even when beneficial compensatory mutations balance the loss of fitness due to Muller's ratchet. Moreover, fitness class fluctuations and the ratchet are arguably maximized in *neutral* populations [52], which are well-characterized by the neutral coalescent. Instead, our results show that we can capture many aspects of silent site diversity simply by correcting for the *average bias* in the fitness distribution away from the prediction in Eq. (1), similar to the findings of Ref. [47]. In order to predict this bias from first principles, it is crucial to account for correlations in the frequencies of selected mutations, similar to rapidly adapting populations [44], [65].

Of course, the degree of interference in any particular organism is ultimately an empirical question — one that hinges on the relative strengths of mutation, selection, and recombination. Although interference is often observed in microbes and viruses [76]–[79], its prevelance in higher sexual organisms is still controversial because it is difficult to estimate these parameters in the wild. Mutation and recombination rates can be measured directly (at least in principle), but population sizes and selection strengths can only be *inferred* from a population genetic model, and these have historically struggled to include the effects of selection on linked sites. Many estimates of “
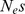
” ignore linkage by fiat (e.g. [80]) under the assumption that sites evolve independently. But these estimates become unreliable precisely when small- and intermediate-effect mutations are most common, and the reasons for this are apparent from Figure 4. All of the distortions in Figure 4 C and Figure 4 D would be mistakenly ascribed to demography (or in the case of Figure 4 E, population substructure), thereby biasing the estimates of selection at nonsynonymous sites. At best, these estimates of “
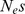
” represent measurements of 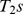, which carry little information about the true strength of selection (*Ns*) or even the potential severity of interference. For example, all of the populations in Figure 8 have *Ns* = 10 and 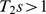, even though they fall in the interference selection regime, and show a strong distortion in minor allele frequency that cannot be explained by Eq. (2). In other words, we cannot conclude that interference is negligible just because “
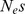
”, as inferred from data, is larger than one.

More sophisticated analyses avoid these issues with simulations of the underlying genomic model [7], [22], [29], [30]. In principle, this approach can provide robust estimates of the underlying parameter combinations that best describe the data. But in practice, simulation-based methods suffer from two major shortcomings which are highlighted by the symmetry above. We have seen that strongly-interfering populations with the same variance in fitness possess nearly identical patterns of genetic diversity. This suggests a degree of “sloppiness” [81] in the underlying model, which can lead to large intrinsic uncertainties in the parameter estimates and a strong sensitivity to measurement noise. A more fundamental problem is identifying the nearly equivalent populations in the first place. Even in our simplified model, large genomes are computationally expensive to simulate, and this obviously limits both the number of dependent variables and the various parameter combinations that can be explored in a single study. We have shown that sets of equivalent populations lie along a single line (namely, the line of constant *Nσ*) in the larger parameter space, which can easily be missed in a small survey unless the parameters are chosen with this degeneracy in mind. In this way, our theoretical predictions can aid existing simulation methods by identifying equivalent sets of parameters that also describe the data.

As an example, we consider the *D. melanogaster* dot chromosome that inspired the parameter combination in Figure 4 A. Earlier, we showed that the reduction in silent site diversity on this chromosome (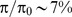) is consistent with the parameters *Ns*≈30, *NU*≈300, and *NR*≈0, which fall in the middle of the interference selection regime (Ref. [29], see Methods). Our calculations allow us to predict other parameter combinations with the same patterns of diversity, and we plot the simulated frequency spectrum for three of these alternatives in Figure 6. We see that even with highly resolved frequency spectra (unavailable in the original dataset), there is little power to distinguish between these predicted alternatives despite rather large differences in the underlying parameters.

However, this “resolution limit” suggests that individual fitness effects are not the most interesting quantity to measure when interference is common. Individual fitness effects may play a central role in single-site models, but we have shown that global properties like the variance in fitness and the corresponding linkage scale are more relevant for predicting evolution in interfering populations. Estimating these quantities directly may therefore be preferable in practice. Our coarse-grained predictions provide a promising new framework for inferring these quantities based on allele frequency data or genealogical reconstruction. A concrete implementation presents a number of additional challenges, mostly to ensure a proper exploration of the high-dimensional parameter space, but this remains an important avenue for future work.

Finally, our findings suggest a *qualitative* shift in the interpretations gleaned from previous empirical studies. We have provided further evidence that even weak purifying selection, when aggregated over a sufficiently large number of sites, can generate strong deviations from neutrality. Moreover, these signals can resemble more “biologically interesting” scenarios like recurrent sweeps, large-scale demographic change, or selection on the silent sites themselves. Here we refer not only to the well-known reduction in diversity and skew towards rare alleles, but also to the topological imbalance in the genealogy (or the “U-shaped” frequency spectrum), and the strong correlations in these quantities with the rate of recombination. Since weakly deleterious mutations are already expected to be common [60], they may constitute a more parsimonious explanation for observed patterns of diversity unless they can be rejected by a careful, quantitative comparison of the type advocated above. At the very least, these signals should not be interpreted as *prima facie* evidence for anything more complicated than weak but widespread purifying selection.

## Methods

### Forward-time simulations

Forward-time simulations were implemented in a custom C++ program using a discrete-generation Wright-Fisher algorithm. Each simulation started with a clonal population of *N* = 10^4^ individuals with initial fitness *W* = 1, and subsequent generations were obtained by performing a reproduction step, a recombination step, and a mutation step. In the reproduction step, the new generation was formed by sampling individuals with replacement from the previous generation, weighted by the relative fitnesses 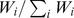. In the recombination step, we drew Poisson(*NR*) recombination events, and for each of these, we drew two individuals from the population and replaced the first individual with the recombinant offspring formed from a single randomly chosen crossover of the two chromosomes. Finally, in the mutation step, we drew Poisson(*NU*) nonsynonymous mutations, and for each of these, we drew an individual from the population and placed the mutation at a random location on the chromosome. The fitness effect of each mutation was drawn from the distribution of fitness effects, *ρ*(*s*), so that the fitness of the mutated individual was given by 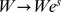. Mutations at the neutral locus were handled similarly, except that these occurred with rate 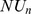 and were always placed at the exact center of the chromosome so that they could not recombine with each other. Starting at generation *t* = 0, each population was allowed to “burn-in” for Δ*t* generations until the neutral locus developed a most recent common ancestor. After equilibration, we drew 100 independent samples of *n* individuals every Δ*t* generations, and the site frequency spectrum was computed at the neutral locus. We also measured the average fitness of the population and computed the variance in fitness using Fisher's fundamental theorem, 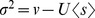, where *v* is the rate of fitness change (e.g., due to Muller's ratchet) which is estimated by 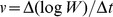. This process was continued for a total of 20*N* generations per population, and for 300 independent populations per parameter combination.

### Coalescent simulations

Backward-in-time simulations of the asexual structured coalescent, the recombining structured coalescent, and the Bolthauzen-Sznitman coalescent were implemented as a set of custom C++ programs similar to Hudson's ms [82]. To improve performance, neutral mutations were omitted, and the time to the next event was replaced with its expected value when calculating the average site frequency spectrum. Asexual coalescent simulations were evaluated 10^5^ times for each parameter value, while the more computationally-demanding recombinant version was evaluated 10^4^ times per parameter value.

### The boundary between the interference and background selection regimes

The boundary of the background selection regime was obtained by minimizing Eq. (3) as a function of *Ns* with 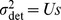 held fixed. Numerical solutions were obtained by analytically differentiating Eq. (3) and inverting the stationarity condition using the Newton-Raphson algorithm in the SciPy library. See Text S3 for additional discussion.

### Coarse-grained predictions

The coarse-grained parameters were obtained by calculating *Nσ* (as described in Text S2) and identifying the corresponding point on the boundary of the interference selection regime with the same value of *Nσ* (as described above). Coarse-grained predictions were obtained from structured coalescent simulations of the coarse-grained parameters, except for 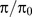, which was approximated by numerical evaluation of Eq. (3).

### Determination of the effective linkage scale

The effective linkage scale, 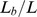, was obtained by inverting the condition
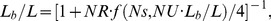
(9)where 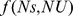 denotes the coarse-grained prediction for 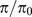 in Eq. (3). Numerical solutions were obtained using the Brent algorithm in the SciPy library. See Text S4 for additional discussion.

### Code availability

We have implemented the methods described above as a Python library, coarse_coal, which can be used to calculate coarse-grained parameters and frequency spectrum predictions for arbitrary combinations of *Ns*, *NU*, and *NR* in the interference selection regime. Our source code is available for download at https://github.com/benjaminhgood/coarse_coal.

### The *Drosophila* dot chromosome

Possible parameter combinations for the fourth (dot) chromosome of *Drosophila melanogaster* were obtained by reapplying the method of Ref. [29] for our simple purifying selection model. These authors estimated the reduction in diversity on the dot chromosome to be 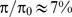, based on sequence data containing approximately *L*∼5 kb of silent sites sequenced in each of *n*≈24 lines [83], [84]. The per-site heterozygosity is of order 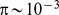, which implies a silent mutation rate of 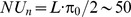. Based on these estimates for the sample size and 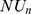, forward-time simulations of the parameters *Ns* = 30, *NU* = 300, and *NR* = 0 yield 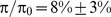 (mean ± s.d.), which is consistent with the observed reduction.

### Human autosomal diversity

Local recombination rates in Figure S6 were estimated from deCODE's fine-scale genetic map [74], assuming an equal sex ratio and averaging over 1 Mb windows. The local mutation rate was approximated using a uniform point-mutation rate of 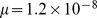 per base pair per generation [85]. Average minor allele frequencies were estimated using the African SNPs identified in the low-coverage portion of the 1,000 Genomes Project [86]. We only included autosomal SNPs that fell within one of the 1 Mb windows identified above, and we excluded repetitive elements (RepeatMasker), RefSeq exons, and all SNPs that were absent or fixed within the African subpopulation or did not have a high-confidence ancestral allele.

## Supporting Information

Code S1 Associated source code.
(ZIP)

Figure S1The breakdown of the structured coalescent. The emergence of the interference selection regime for a recombining genome with *U*/*R*∼1, as measured by the reduction in silent site heterozygosity (top) and the average minor allele frequency from a sample of size *n* = 100 (middle). Symbols denote forward-time simulations of our simple purifying selection model, while the predictions from the structured coalescent and the background selection limit are represented by the solid and dashed lines, respectively. For comparison, the bottom panel shows a measure of the linkage disequilibrium between selected mutations, as measured by the quantity 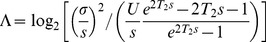.(PNG)

Figure S2Full site frequency spectra from Figure 3. The silent site frequency spectrum for each of the simulated populations in Figure 3, noramlized by the the number of singletons (top) or *π* (bottom). Colored lines are measured from a sample of *n* = 100 chromosomes, averaged over independent populations (see Methods). For comparison, the solid black line shows the neutral expectation, while the dotted line shows the 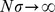 limit from Ref. [44]. In the interference selection regime (right), the shape of the frequency spectrum is strongly correlated with the reduction in pairwise diversity, 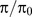. This is a manifestation of the infinitesimal limit, where both quantities are controlled by *Nσ*. In contrast, the correlation disappears in the background selection regime (left) as predicted by the structured coalescent.(PNG)

Figure S3
Figure 4 replotted for the background selection regime. Distortions in the synonymous site frequency spectrum for a sample of *n* = 100 individuals in the background selection regime. Top: An excess of rare alleles measured by the average minor allele frequency. Middle: Tajima's *D*. Bottom: Non-monotonic or “U-shaped” behavior at high frequencies, as measured by 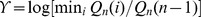. Both statistics are plotted as a function of the reduction in pairwise diversity, 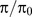. Upper triangles depict the subset of simulations in Figure 3 that were classified into the background selection regime, and each point is colored according to its *Ns* value. For comparison, the dashed blue lines show the predictions in the background selection limit, which coincide with the neutral expectation.(PNG)

Figure S4The reduction in pairwise diversity at silent sites for three different distributions of deleterious fitness effects. Colored symbols denote the results of forward time simulations for asexual populations with 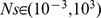 and 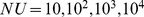. We performed simulations for three DFEs: a single-*s* distribution with 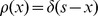, a uniform distribution with 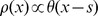, and a truncated exponential distribution with 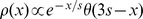. 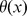 is the step function. Each point is colored according to its 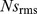 value. For comparison, our coarse-grained predictions are shown in solid red while the dashed lines show the neutral expectation.(PNG)

Figure S5Genetic diversity in a “hybrid” two-effect model. The reduction in silent site heterozygosity (top) and the average minor allele frequency from a sample of size *n* = 100 (middle) in a two-effect model with one weakly deleterious mutation (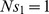, 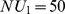) and one strongly deleterious mutation (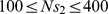). Black symbols denote the results of forward-time simulations where 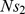 is increased from 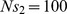 to 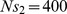, while the product 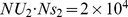 is held constant. For comparison, the bottom panel shows the measured variance in fitness. Our coarse-grained predictions are shown in solid red throughout, while the two-effect generalization of the structured coalescent is shown in solid blue.(PNG)

Figure S6Recombination rates in human autosomes. Top: the distribution of “mutation density” (i.e., the ratio *U*/*R*) along the human autosomes. Local recombination rates were estimated from the deCODE genetic map [74] and averaged over 1 Mb windows (Methods), and we assume a uniform point-mutation rate of 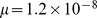 per base pair [85]. Bottom: the average African minor allele frequency estimated by the 1,000 Genomes Project [86] (Methods).(PNG)

Text S1 Background selection and the structured coalescent.
(PDF)

Text S2 The infinitesimal limit.
(PDF)

Text S3 The coarse-grained coalescent.
(PDF)

Text S4 Recombining genomes.
(PDF)
