# Supplementary figures and images for: Genetic Diversity in the Interference Selection Limit
Source: PLoS Genet. 2014 Mar 27;10(3):e1004222. doi: 10.1371/journal.pgen.1004222 (PMC3967937; doi:10.1371/journal.pgen.1004222)

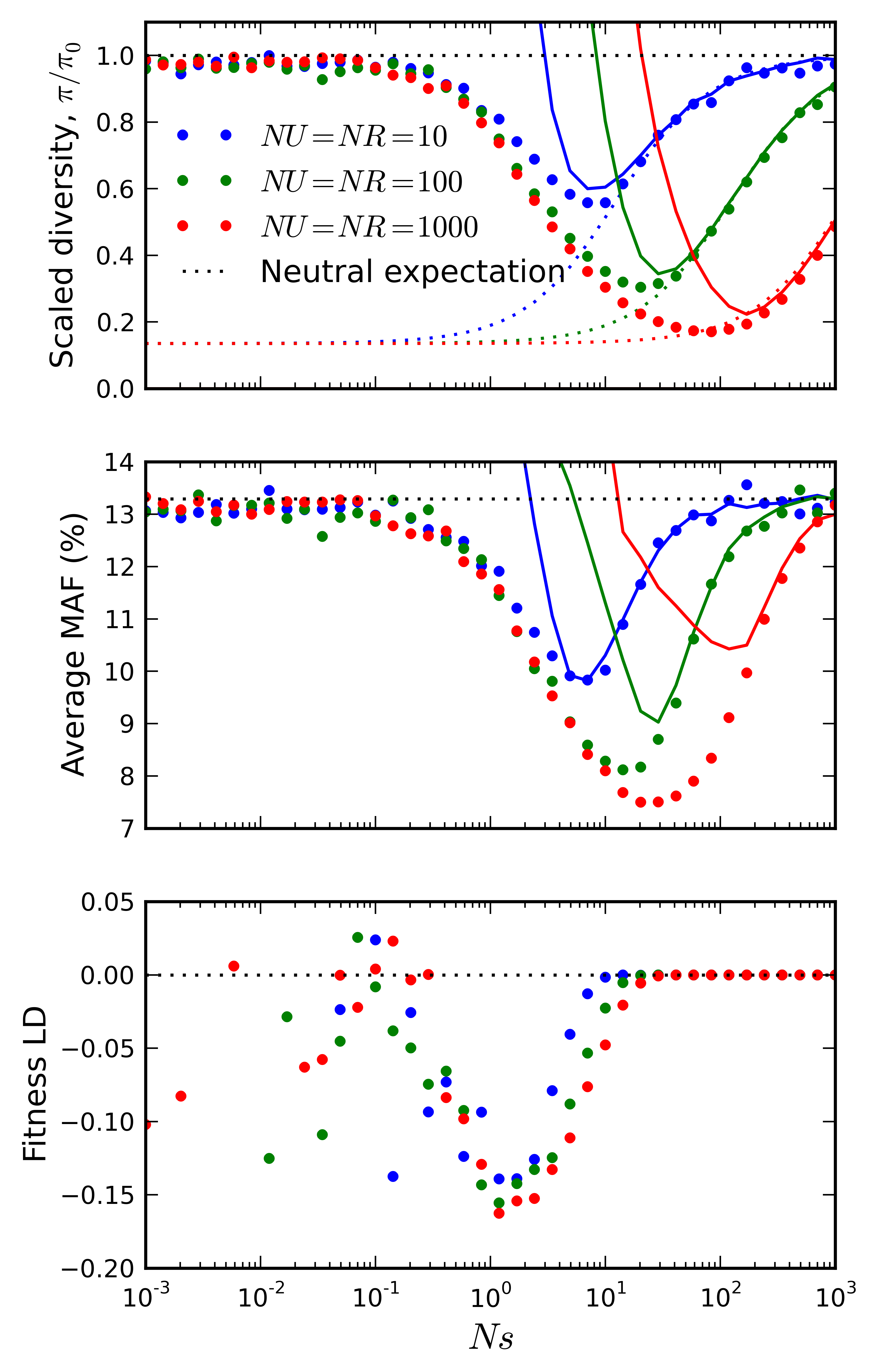

Supplement: Figure S1 — The breakdown of the structured coalescent. The emergence of the interference selection regime for a recombining genome with U/R∼1, as measured by the reduction in silent site heterozygosity (top) and the average minor allele frequency from a sample of size n = 100 (middle). Symbols denote forward-time simulations of our simple purifying selection model, while the predictions from the structured coalescent and the background selection limit are represented by the solid and dashed lines, respectively. For comparison, the bottom panel shows a measure of the linkage disequilibrium between selected mutations, as measured by the quantity . (PNG) [file pgen.1004222.s002.png]

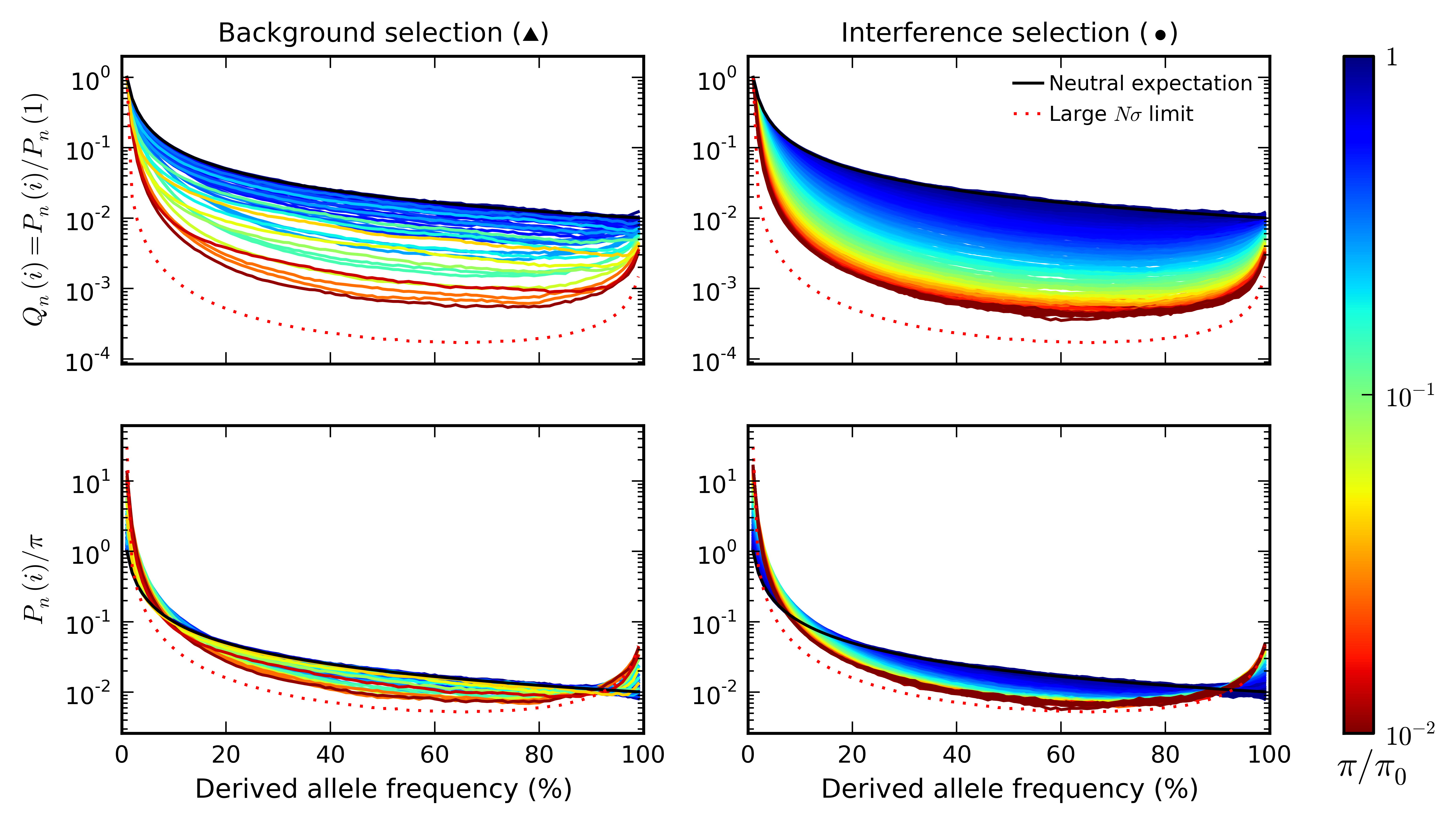

Supplement: Figure S2 — Full site frequency spectra from Figure 3. The silent site frequency spectrum for each of the simulated populations in Figure 3, noramlized by the the number of singletons (top) or π (bottom). Colored lines are measured from a sample of n = 100 chromosomes, averaged over independent populations (see Methods). For comparison, the solid black line shows the neutral expectation, while the dotted line shows the limit from Ref. [44]. In the interference selection regime (right), the shape of the frequency spectrum is strongly correlated with the reduction in pairwise diversity, . This is a manifestation of the infinitesimal limit, where both quantities are controlled by Nσ. In contrast, the correlation disappears in the background selection regime (left) as predicted by the structured coalescent. (PNG) [file pgen.1004222.s003.png]

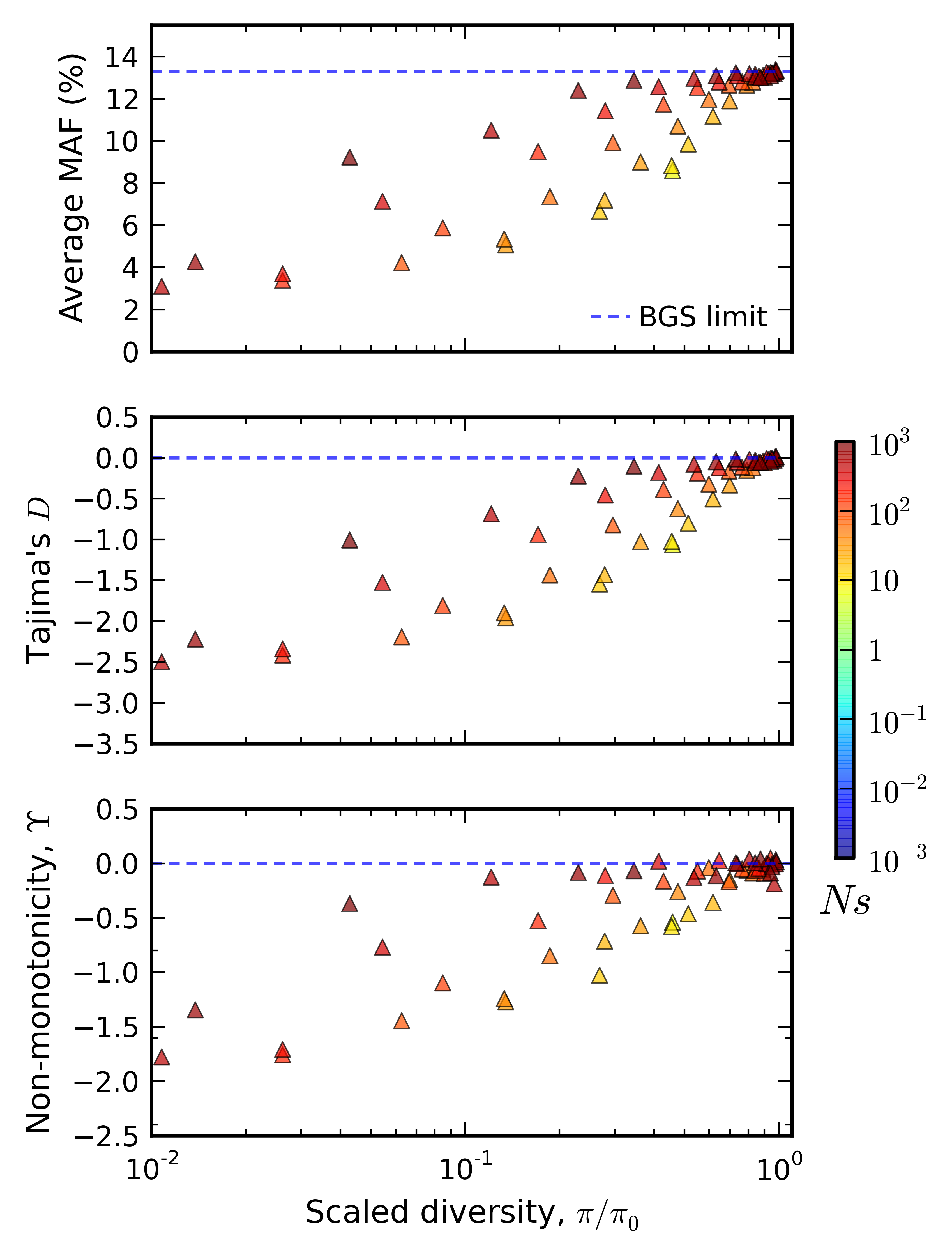

Supplement: Figure S3 — Figure 4 replotted for the background selection regime. Distortions in the synonymous site frequency spectrum for a sample of n = 100 individuals in the background selection regime. Top: An excess of rare alleles measured by the average minor allele frequency. Middle: Tajima's D. Bottom: Non-monotonic or “U-shaped” behavior at high frequencies, as measured by . Both statistics are plotted as a function of the reduction in pairwise diversity, . Upper triangles depict the subset of simulations in Figure 3 that were classified into the background selection regime, and each point is colored according to its Ns value. For comparison, the dashed blue lines show the predictions in the background selection limit, which coincide with the neutral expectation. (PNG) [file pgen.1004222.s004.png]

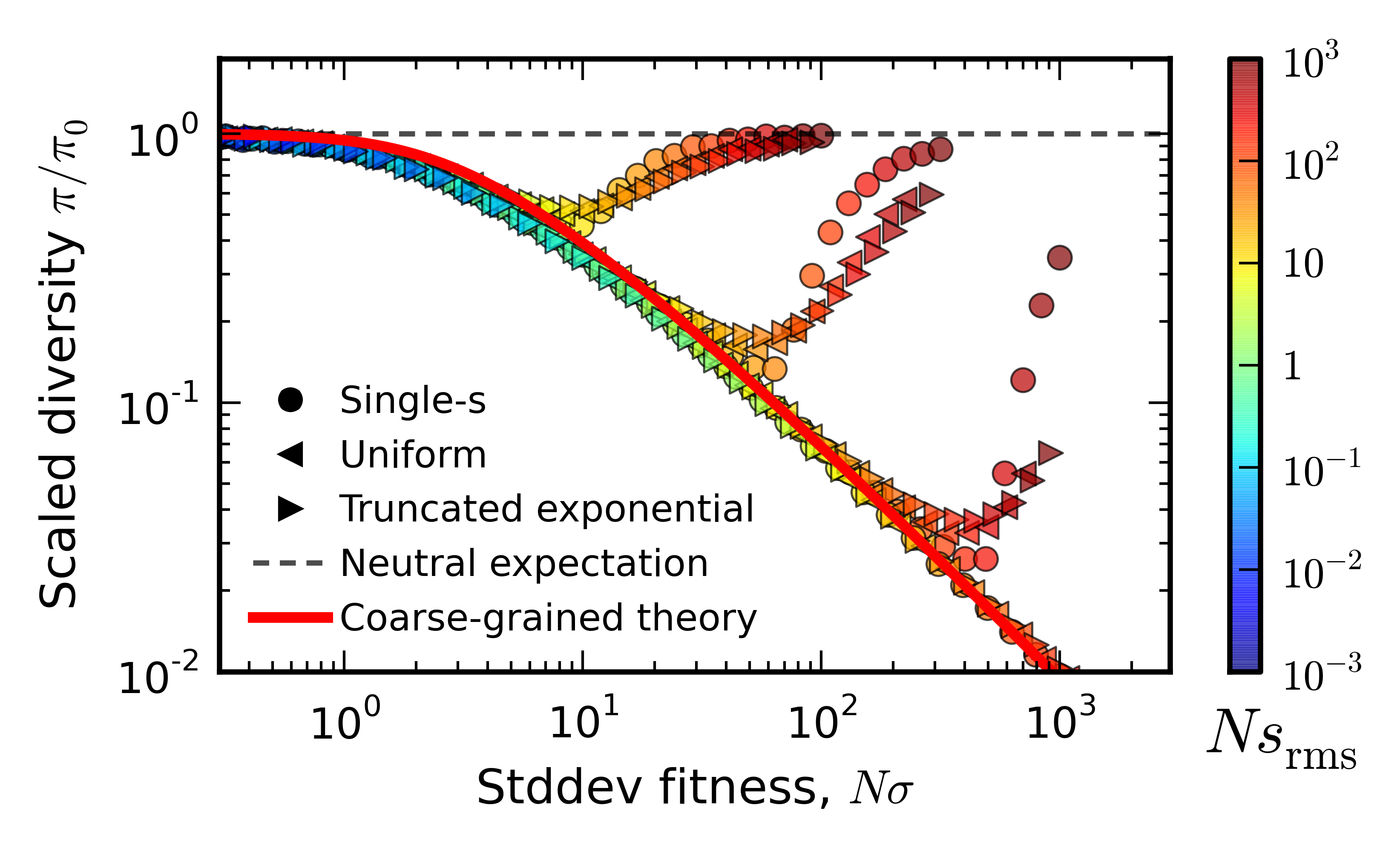

Supplement: Figure S4 — The reduction in pairwise diversity at silent sites for three different distributions of deleterious fitness effects. Colored symbols denote the results of forward time simulations for asexual populations with and . We performed simulations for three DFEs: a single-s distribution with , a uniform distribution with , and a truncated exponential distribution with . is the step function. Each point is colored according to its value. For comparison, our coarse-grained predictions are shown in solid red while the dashed lines show the neutral expectation. (PNG) [file pgen.1004222.s005.png]

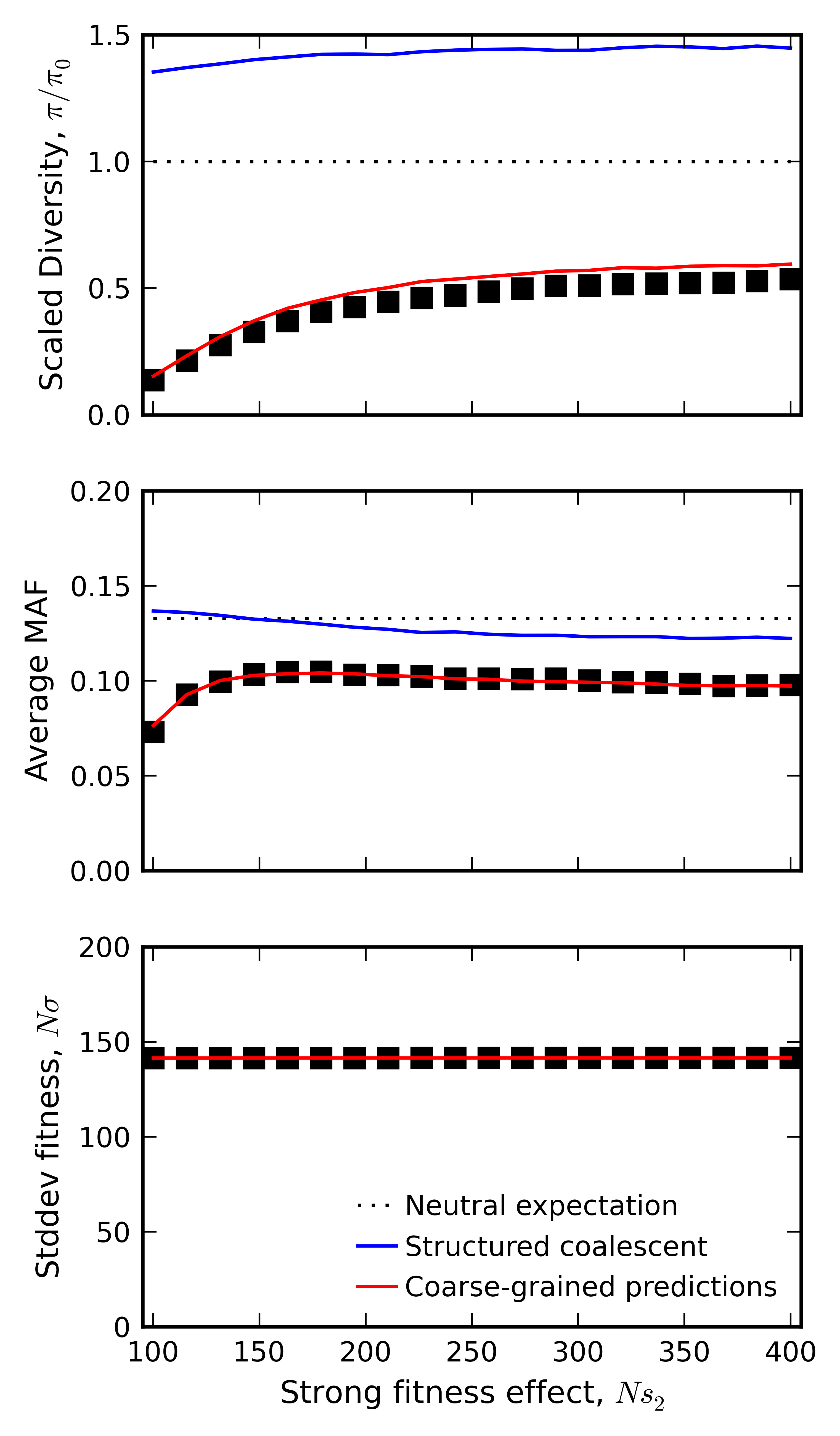

Supplement: Figure S5 — Genetic diversity in a “hybrid” two-effect model. The reduction in silent site heterozygosity (top) and the average minor allele frequency from a sample of size n = 100 (middle) in a two-effect model with one weakly deleterious mutation (, ) and one strongly deleterious mutation (). Black symbols denote the results of forward-time simulations where is increased from to , while the product is held constant. For comparison, the bottom panel shows the measured variance in fitness. Our coarse-grained predictions are shown in solid red throughout, while the two-effect generalization of the structured coalescent is shown in solid blue. (PNG) [file pgen.1004222.s006.png]

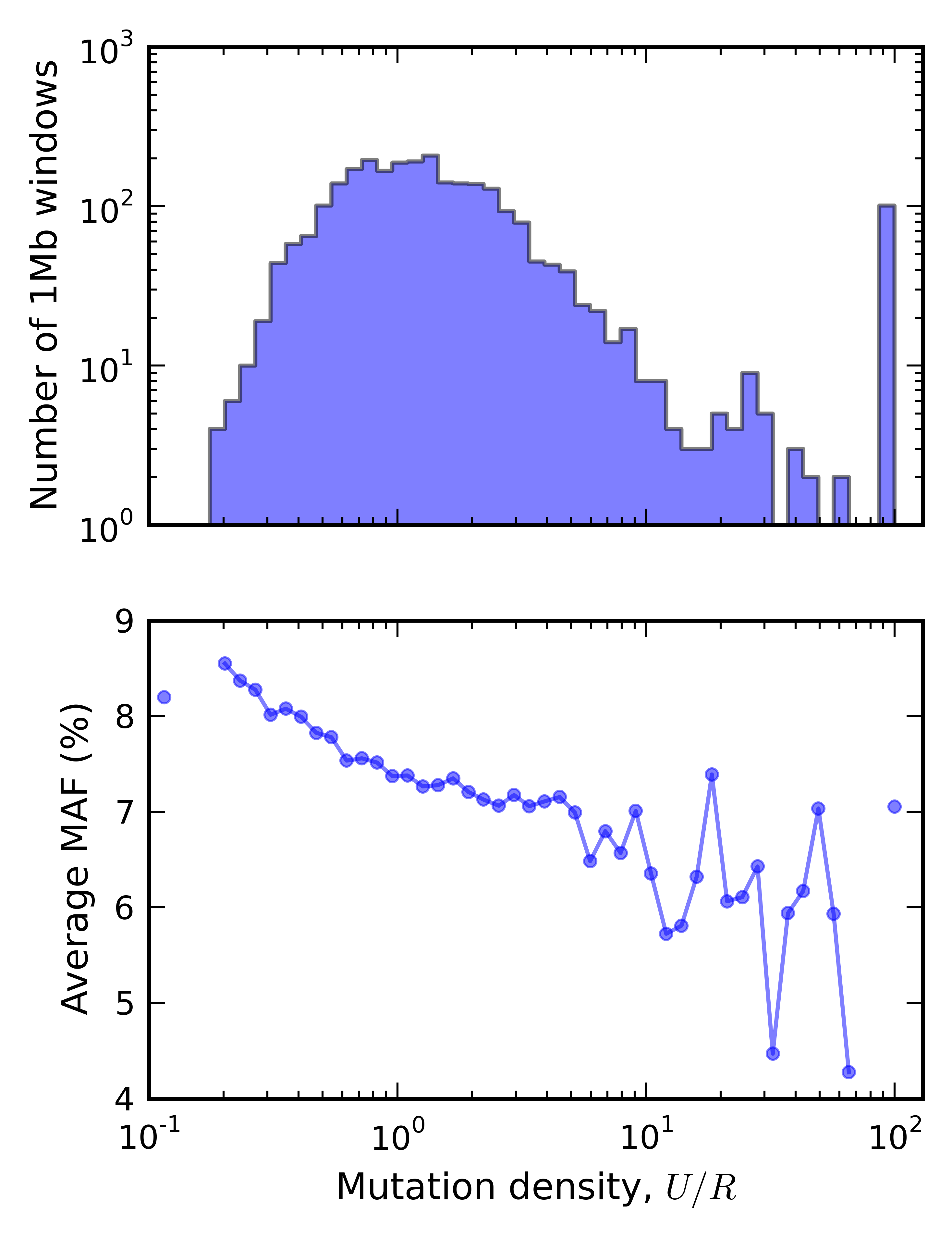

Supplement: Figure S6 — Recombination rates in human autosomes. Top: the distribution of “mutation density” (i.e., the ratio U/R) along the human autosomes. Local recombination rates were estimated from the deCODE genetic map [74] and averaged over 1 Mb windows (Methods), and we assume a uniform point-mutation rate of per base pair [85]. Bottom: the average African minor allele frequency estimated by the 1,000 Genomes Project [86] (Methods). (PNG) [file pgen.1004222.s007.png]
