# Supplementary material for: Genetic Diversity in the Interference Selection Limit
Source: PLoS Genet. 2014 Mar 27;10(3):e1004222. doi: 10.1371/journal.pgen.1004222 (PMC3967937; doi:10.1371/journal.pgen.1004222)
Supplement: Text S1 — Background selection and the structured coalescent. (PDF) [file pgen.1004222.s008.pdf]

# Text S1 Background selection and the structured coalescent

The background selection limit in Eq. (2) and the structured coalescent corrections have been studied for more than two decades [16, 40], and the resulting behavior has been well characterized. Nevertheless, our present analysis suggests a rather different interpretation of the background selection limit and the structured coalescent corrections than is usually cited in the literature. Since our coarse-grained predictions in Text S3 will strongly depend on this existing theory, we will use the present section to precisely define the background selection limit and try to dispel any misconceptions about this regime that could cause confusion later on. For the moment, we restrict our attention to the asexual ($R \approx 0$) limit, while recombining genomes are treated in Text S4.

## The background selection limit

Although it is not typically motivated in this fashion, the background selection limit in Eq. (2) emerges as a formal limit of the evolutionary model in Eq. (4). The limit is one in which $Ns \to \infty$ and $NU \to \infty$ with the ratio $\lambda = U/s$ held fixed, in which case the patterns of neutral diversity are equivalent to a neutral population with an effective population size $N_e = Ne^{-\lambda}$. Thus, an equivalent statement of this limit is that $N_e s \to \infty$ with $N_e/N$ fixed. If all mutations share the same fitness effect, the distribution of fitnesses can be calculated from Eq. (4) by suppressing the mutant and wildtype indices. Rescaling time by $\tau = t/N_e$ and fitness by $k = X/s$, we obtain the Langevin equation

$$\begin{aligned}\frac{\partial f(k)}{\partial \tau} &= Nse^{-\lambda}\left[k - \overline{k}(t)\right] f(k) + NUe^{-\lambda}\left[f(k-1) - f(k)\right] \\ &\quad + \sum_{k'} \left[\delta_{kk'} - f(k)\right] \sqrt{f(k)}\eta(k)\,,\end{aligned} \tag{ST1.1}$$

where $k$ counts the number of deleterious mutations in each individual [50]. When $N_e s \to \infty$, a self-consistent solution can be obtained by dropping the time-derivative and the stochastic drift terms, which yields the deterministic mutation-selection balance in Eq. (1), originally derived in Ref. [32]. This implies that the effective population size above is equal to the number of mutation-free individuals in the population. The average number of mutations in each individual (i.e., the average deleterious load) is given by $\lambda = U/s$, and the variance in fitness within the population is $\sigma^2_{\mathrm{det}} = Us$. Moreover, since $f(k)$ follows a Poisson distribution in this limit, we would have obtained the same result by solving for mutation selection balance at each site independently and assuming that these sites were in linkage equilibrium. In other words, the background selection limit is one in which *deleterious mutations* do not interfere with each other except in the most trivial sense through a reduction in $N_e$. These mutations behave as if they were in linkage equilibrium (despite the fact that they are *actually* linked) because selection is so strong. Deleterious mutations are purged so quickly that one rarely encounters two individuals with the *same* deleterious mutation, even though new mutations are created so often that a large fraction of the population possess *some* deleterious mutations. In other words, all deleterious mutations are private.

This leads to the following genealogical interpretation, which is illustrated in Figure 1 in the main text. A sample of individuals is drawn from the population, and the number of deleterious mutations in each individual is given by independent draws from Eq. (1). All of these mutations are private (i.e., they will be located on external branches), which implies that coalescent events cannot happen until the ancestors of the sample are free of deleterious mutations. Since selection is so strong, this happens almost instantaneously, after which point the lineages are free to coalesce neutrally with an effective population size $N_e = Ne^{-\lambda}$ given by the number of mutation-free individuals.

## The structured coalescent

Of course, selection strengths are never *actually* infinite, and it is well-known that Eq. (2) fails to capture certain aspects of molecular evolution when $N_e s < \infty$. This is particularly evident in the silent site frequency spectrum in Figure S3, where significant deviations from neutrality can be observed even when $\pi$ is well-approximated by Eq. (2) [39,41]. We can predict these distortions using a *perturbative approach*, which leverages our characterization of the evolutionary dynamics in the background selection limit. This is based on the idea that for large (but finite) $N_e s$, non-neutral distortions will arise as (initially small) corrections to Eq. (2). Formally, this is equivalent to the statement that every diversity statistic $D$ can be expanded in an asymptotic series of the form

$$D \sim D_{bgs} + \frac{g(\lambda)}{Ns} + \text{higher order terms}\,; \quad (Nse^{-\lambda} \to \infty)\,, \tag{ST1.2}$$

where $D_{bgs}$ is the value of that statistic in the background selection limit. For example, if $D = T_2/N$ then $D_{bgs} = e^{-\lambda}$ and $g(\lambda)/Ns$ represents a small fractional correction to the background selection value. Even though the corrections are small, we can in principle construct differences of the form

$$(D - D_{bgs})Ns \sim g(\lambda) + \text{higher order terms}\,; \quad (Nse^{-\lambda} \to \infty)\,, \tag{ST1.3}$$

which remain finite even in the $Nse^{-\lambda} \to \infty$ limit. In general, contributions to $g(\lambda)$ and other higher-order terms can arise from several sources:

1. corrections to the average shape of the deterministic mutation-selection balance in Eq. (1).
2. stochastic fluctuations around the steady-state in Eq. (1).
3. fixation of deleterious mutations via Muller's ratchet, and the subsequent (irreversible) translation of Eq. (1) toward higher $k$.
4. coalescence events that occur before all of the ancestors of the sample are free of deleterious mutations.
5. finite relaxation time for ancestral lineages to move through the fitness distribution.

The structured coalescent is a method for calculating genealogical corrections to the background selection limit that focuses solely on effects (4) and (5), while neglecting the others.[1] In particular, it assumes that the fitness classes are fixed at the deterministic mutation-selection balance in Eq. (1), and it concentrates on movement (mutation) and coalescence within this fixed structure. Under this assumption, the coalescent process is equivalent to a demographically structured *neutral* population, where the fitness classes denote different demes and deleterious mutations are recast as migration events between them.

The structured coalescent provides a probabilistic model for the genealogy of $n$ randomly selected individuals from such a population. Since they are initially sampled at random, these individuals come from from fitness classes $k_1, \ldots, k_n$ drawn from the population fitness distribution in Eq. (1), which implies that

$$k_1, \ldots, k_n \overset{i.i.d.}{\sim} \text{Poisson}\,(\lambda)\,. \tag{ST1.4}$$

We then trace the genealogy back to the most recent common ancestor of the sample. At any given instant, two types of ancestral events can occur:

[1] In its most general form, the structured coalescent extends the Kingman coalescent to include arbitrary time-dependent (and possibly stochastic) demographic structure [33]. The stochastic version has been used to model the effects of selection at a single locus [43], but it is difficult to generalize these results when many sites are under selection. In the present work, we focus our attention on the approximation proposed by Ref. [40], where the relevant demographic structure is the division of the population into constant fitness classes attained at mutation-selection balance.

1. An individual in class $k_i > 0$ can experience a deleterious mutation (thus transferring it to class $k_i - 1$) at rate
$$\frac{UNf(k_i-1)}{Nf(k_i)} = sk_i \,. \tag{ST1.5}$$
2. Two individuals in the same fitness class $k = k_i = k_j$ can coalesce to a single individual at rate
$$Nf(k)\cdot\left(\frac{1}{Nf(k)}\right)^2 = \frac{1}{Nf(k)} \,. \tag{ST1.6}$$

These events form a competing Poisson process, which implies that the time to the next event is exponentially distributed with mean equal to the reciprocal of the sum of the rates of all possible events. The event itself is then drawn randomly from the pool of possible events weighted by their corresponding rates. This process continues until the sample has coalesced into a single lineage, which is the most recent common ancestor of the sample.

Thus, for a given set of parameters $Ns$ and $NU$, the distribution of genealogies for a sample of $n$ individuals is completely specified. This algorithm has a straightforward implementation as a background-in-time coalescent simulation (similar to Hudson's `ms` [82]), which can be used to sample genealogies from this distribution. The distribution of an arbitrary diversity statistic $D$ can be then be obtained by averaging over the distribution of genealogies. The primary advantage of these coalescent simulations is that they are extremely fast, with a runtime that scales as $\mathcal{O}(\lambda \cdot (n+\lambda))$ compared to the $\mathcal{O}(NU \cdot N)$ time required for forward-time simulations.

Of course, the other advantage of the structured coalescent is that it accurately captures many of the deviations from Eq. (2) for finite $N_e s$, even down to $N_e s \sim 1$ [36,41]. This is a welcome surprise, since we know that the structured coalescent neglects crucial stochastic effects (e.g., fitness class fluctuations and Muller's ratchet) that grow increasingly strong as $N_e s$ becomes smaller. Somewhat confusingly, previous authors have interpreted this numerical agreement as evidence that the structured coalescent captures deviations from Eq. (2) that are "caused" by Muller's ratchet [41]. This is clearly not the case; rather, the structured coalescent explicitly *ignores* fluctuation effects and asks how much of the deviation from Eq. (2) can be explained by variation in ancestral fitness alone. The fact that the *deterministic* structured coalescent seems to reproduce many of the deviations from Eq. (2) is therefore quite interesting, since it suggests that fluctuation effects contribute only minor corrections to the ancestral process when $N_e s \gtrsim 1$, even when the ratchet is nominally operating.

This distinction will later prove to be crucial when we introduce our coarse-grained model for the interference selection regime. Essentially, we will argue that interference alters the steady-state shape of the fitness distribution in Eq. (1), as described in Text S2, but that otherwise the effects of fluctuations and the ratchet can be minimized by sufficient coarse-graining. In other words, we assume that the deviations from neutrality are still largely determined by variation in ancestral fitness, similar to the semi-analytical approach in Ref. [47]. This represents a significant departure from many previous studies, which argue that stochastic effects are the *defining* feature of the interference selection regime, and that fundamentally new methods are required to predict the diversity in these populations [22,43].

Finally, it is worth noting that while the numerical agreement between forward-time simulations and the structured coalescent is promising, this accuracy is difficult to justify from a formal standpoint. For example, the first-order corrections to $T_2/N$ from the structured coalescent arise at order $\mathcal{O}(N_e s)^{-1}$, but it is also known that variance in the number of mutation-free individuals enters at the same order in $N_e s$ [50]. (Muller's ratchet, on the other hand, is exponentially suppressed.) Thus, there is technically no asymptotic limit in which the effects of fluctuations can be made arbitrarily small compared to the structured coalescent corrections. The structured coalescent predictions for $g(\lambda)$ will therefore be inaccurate even in the limit that $N_e s \to \infty$. Clearly, some fortuitous cancellation of these effects occurs for the moderate values of $N_e s$ that are accessible in forward-time simulations. For our purposes this will be sufficient, but the resolution of these issues remains an important avenue for future work.

## Derivation of Eq. (3)

In this section, we provide a derivation for Eq. (3) in the main text. Ref. [37] has shown that for large $N_e s$, the structured coalescent predictions for $T_2$ are well-approximated by a neutral population with a time-varying population size

$$N_e(t) = N \exp\left[-\lambda \left(1 - e^{-st}\right)^2\right] , \tag{ST1.7}$$

so that the average average value of $T_2$ is given by

$$\begin{aligned} T_2 &= \int_0^\infty dt \, \frac{t}{N_e(t)} \exp\left[-\int_0^t \frac{dt'}{N_e(t')}\right] , \\ &= \int_0^\infty dt \, \exp\left[-\int_0^t \frac{dt'}{N_e(t')}\right] . \end{aligned} \tag{ST1.8}$$

The effective population size from Ref. [37] is part of a larger class of demographic models with a population expansion only over a very recent timescale $T_d \ll T_2$, so that the bulk of the ancestral process is subject to the constant population size

$$N_e(t) \sim N_\infty , \quad t \gg T_d . \tag{ST1.9}$$

In the particular example above, $T_d = 1/s$ and $N_\infty = N e^{-\lambda}$. If $T_d/N_\infty \to 0$, we would simply have $T_2 = N_\infty$, while the correction in Eq. (3) is given by the next term in an asymptotic expansion of the integral in the limit of small $\epsilon = T_d/N_\infty$. To see this, we first rescale Eq. (ST1.8), defining

$$\tau = \frac{t}{N_\infty} , \quad f(\tau) = \left(\frac{N_e(\tau N_\infty)}{N_\infty}\right)^{-1} , \tag{ST1.10}$$

where $f(\tau)$ possesses the asymptotic properties

$$f(\xi) \sim 1 , \quad f'(\epsilon\xi) \sim \epsilon^{-1} , \tag{ST1.11}$$

when $\epsilon \to 0$ and $\xi \sim \mathcal{O}(1)$. The integral can then be rewritten in the dimensionless form

$$\tau_2 = \int_0^\infty d\tau \exp\left[-\int_0^\tau d\tau' f(\tau')\right] . \tag{ST1.12}$$

In the limit that $\epsilon \to 0$, the behavior of this integrand depends on whether $\tau$ is greater or less than $\epsilon$. To this end, we define $\delta_1 = \epsilon^{2/3}$ so that $\delta_1 \gg \epsilon$, and then we split the integration over $\tau$ into the intervals $(0, \delta_1)$ and $(\delta_1, \infty)$, so that

$$\tau_2 = \underbrace{\int_0^{\delta_1} d\tau \exp\left[-\int_0^\tau d\tau' f(\tau')\right]}_{A} + \underbrace{\int_{\delta_1}^\infty d\tau \exp\left[-\int_0^\tau d\tau' f(\tau')\right]}_{B} . \tag{ST1.13}$$

To calculate $A$, we switch variables from $\tau$ to $\xi = \tau/\delta_1$, which shows that

$$\begin{aligned} A &= \delta_1 \int_0^1 d\xi \exp\left[-\delta_1 \int_0^\xi d\xi \, f(\delta_1 \xi)\right] , \\ &= \delta_1 + \mathcal{O}(\delta_1)^2 . \end{aligned} \tag{ST1.14}$$

To calculate $B$, we first focus on the integral inside the exponential, $C(\tau, \lambda, \epsilon) \equiv \int_0^\tau d\tau' \, f(\tau')$, where $\tau \geq \delta_1 \gg \epsilon$. Again, the basic behavior of the integrand depends on whether $\tau'$ is greater or less than $\epsilon$. To this end, we define another scale $\delta_2 = \epsilon^{3/4}$, so that $\tau \geq \delta_1 \gg \delta_2 \gg \epsilon$, and then we split the region of integration into the intervals $(0, \delta_2)$ and $(\delta_2, \tau)$, so that

$$C(\tau, \lambda, \epsilon) = \underbrace{\int_0^{\delta_2} d\tau' \, f(\tau')}_{C_1} + \underbrace{\int_{\delta_2}^{\tau} d\tau' \, f(\tau')}_{C_2} \, . \tag{ST1.15}$$

To calculate $C_1$, we switch variables from $\tau'$ to $\xi = \tau'/\epsilon$ and integrate by parts to obtain

$$C_1 \approx \delta_2 - \epsilon \int_0^\infty d\xi \, \xi \, [f'(\epsilon\xi)\epsilon] \, , \tag{ST1.16}$$

while it is easy to see that $C_2 \approx \tau - \delta_2$. Thus, the total contribution from the $B$ term in Eq. (ST1.14) is given by

$$\begin{aligned} B &= \int_{\delta_1}^\infty d\tau \exp\left[-\tau + \epsilon \int_0^\infty d\xi \, \xi \, [f'(\epsilon\xi)\epsilon]\right] \, , \\ &\approx 1 - \delta_1 + \epsilon \int_0^\infty d\xi \, \xi \, [f'(\epsilon\xi)\epsilon] \, , \end{aligned} \tag{ST1.17}$$

and

$$\tau_2 = 1 + \epsilon \int_0^\infty d\xi \, \xi \, [f'(\epsilon\xi)\epsilon] + o(\epsilon) \, . \tag{ST1.18}$$

Substituting in our specific expressions for $\epsilon$ and $f(\tau)$,

$$\epsilon = \frac{1}{Nse^{-\lambda}} \, , \quad f(\tau) = \exp\left[-2\lambda e^{-\tau/\epsilon} + \lambda e^{-2\tau/\epsilon}\right] \, , \tag{ST1.19}$$

we obtain

$$\frac{\pi}{\pi_0} \equiv \tau_2 e^{-\lambda} = e^{-\lambda} + \frac{2\lambda e^{-\lambda}}{Ns} \int_0^1 \xi \log\left(\frac{1}{1-\xi}\right) e^{\lambda\xi^2} \, d\xi \, + o(Ns)^{-1} \, , \tag{ST1.20}$$

as desired. It is also straightforward to extend this calculation to the variable-$s$ or recombinant forms of $N_e(t)$ derived in Ref. [38].
