# Supplementary material for: Genetic Diversity in the Interference Selection Limit
Source: PLoS Genet. 2014 Mar 27;10(3):e1004222. doi: 10.1371/journal.pgen.1004222 (PMC3967937; doi:10.1371/journal.pgen.1004222)
Supplement: Text S2 — The infinitesimal limit. (PDF) [file pgen.1004222.s009.pdf]

Text S2 The infinitesimal limit

In this section we present a derivation of the infinitesimal limit for non-recombining genomes. As defined in the text, this limit corresponds to $NU \to \infty$ and $N\sqrt{\langle s^2 \rangle} \to 0$ with the product $N\sigma_0 = \sqrt{N^3 U \langle s^2 \rangle}$ held fixed. We will show that the population fitness distribution and the dynamics of a mutant allele depend only on the control parameter $N\sigma_0$ in this limit. We will work with a generalized version of the Langevin dynamics in Eq. (4),

$$\begin{aligned}\frac{\partial f_i(X)}{\partial t} &= \left[X - \overline{X}(t)\right] f_i(X) + U \int ds\, \rho(s)\, [f_i(X-s) - f_i(X)] \\ &\quad + \frac{1}{\sqrt{N}} \sum_j \int dX' \left[\delta_{ij}\delta(X'-X) - f_i(X)\right] \sqrt{f_j(X')}\eta_j(X')\,,\end{aligned} \tag{ST2.1}$$

which includes a general distribution of fitness effects $\rho(s)$.

The basic idea behind the infinitesimal limit is simple. The distribution of fitness effects only enters into the equations through the integral in the mutation term, which can be expanded in a Taylor series,

$$U \int ds\, \rho(s)\, [f(X-s) - f(X)] = \sum_{m=1}^{\infty} \frac{(-1)^m}{m!} U \langle s^m \rangle \partial_X^m f(X)\,, \tag{ST2.2}$$

where we have defined $\langle s^m \rangle = \int s^m \rho(s)\, ds$. Thus, as $Ns \to 0$ we might expect the higher-order moments in this expansion to become negligible in comparison to the lower-order moments. However, why the *second* moment $\langle s^2 \rangle$ (rather than the first moment) remains relevant in the $Ns \to 0$ limit is somewhat more subtle: the argument is that $\langle s \rangle$ only influences absolute fitnesses, so that it disappears when fitness is measured relative to $\overline{X}$. Several derivations are given in the literature [44, 54, 55], though these focus on the distribution of fitnesses within the population and ignore any fluctuations that arise due to drift. Here, we take the opposite approach and present a formal argument that includes all of the stochasticity in the evolutionary process. The basic idea behind the proof will remain the same, but this will allow us to extend these results to the dynamics of sequence evolution as well. Alternatively, one can also derive the infinitesimal limit order-by-order from the perturbation expansion in Ref. [52].

Derivation using generating functions

Following previous studies, we would like to switch into a reference frame where the mean fitness is defined to be zero. If there were no fluctuations and no transient behavior, we could achieve this through a simple Galilean boost $X \to X - vt$ in Eq. (ST2.1), where v is the average rate of adaptation (or fitness decline) in the steady state. However, because the mean fitness is itself stochastic, this task is slightly more complicated. To this end, we define $x = X - \overline{X}$ to be the instantaneous relative fitness, and we focus on the generating function(al)

$$\begin{aligned}H[\phi(x), t] &= \left\langle \exp\left[-\int dx \phi(x) f(x + \overline{X}(t), t)\right]\right\rangle\,, \\ &= \left\langle \exp\left[-\int dX \phi(X - \overline{X}(t)) f(X, t)\right]\right\rangle\,,\end{aligned} \tag{ST2.3}$$

where $f(X,t) = f_0(X,t) + f_1(X,t)$. This generating function encapsulates the full statistics of the fitness distribution at a single time t, ignoring the distinction between the mutant and background types. Similarly, the generating functional

$$G[\phi_0(x), \phi_1(x), t] = \left\langle \exp\left[-\sum_i \int dX \phi_i(X - \overline{X}(t)) f_i(X, t)\right]\right\rangle \tag{ST2.4}$$

gives the full statistics of the mutant and background types and the fitness distribution of their offspring. We recover the generating function for the frequency of the mutant allele (i.e., the central quantity in single-site models) by setting $\phi_0(x) = 0$ and $\phi_1(x) = z$.

To determine the time evolution of H, we must evaluate $H[\phi(x), t + dt]$ through $\mathcal{O}(dt)$ using the Langevin dynamics in Eq. (ST2.1). This yields the following functional differential equation,

$$\begin{aligned}\frac{\partial H}{\partial t} &= \int dx \left[x\phi(x) + \sum_{m=2}^{\infty} \frac{U\langle s^m \rangle \partial_x^m \phi(x)}{m!} - \frac{\phi(x)^2}{2N} - \frac{x\partial_x \phi(x)}{N} \right] \frac{\delta H}{\delta\phi(x)} \\ &+ \int dx\, dy \left[\partial_x \phi(x) y^2 - \frac{\partial_x^2 \phi(x) y^2}{2N} - \frac{\phi(x)\phi(y)}{2N} - \frac{x\phi(x)\partial_y \phi(y)}{N} \right] \frac{\delta H}{\delta\phi(x)\delta\phi(y)} \\ &- \int dx\, dy\, dz \left[\frac{\partial_x \phi(x) \partial_y \phi(y) z^2}{2N} \right] \frac{\delta H}{\delta\phi(x)\delta\phi(y)\delta\phi(z)} \,.\end{aligned} \tag{ST2.5}$$

Thus, we can see that by switching into the instantaneous frame of the mean, all dependence on $\langle s \rangle$ is removed from the equations. In other words, the distribution of *relative* fitness is independent of $\langle s \rangle$, no matter what form $\rho(s)$ takes. Now the lowest-order moment that remains is $U\langle s^2 \rangle$, which should be the only relevant parameter in the $Ns \to 0$ limit. To see this, we rescale time and fitness by

$$\tau = t/N \,, \quad \chi = x/\sqrt{NU\langle s^2 \rangle} \,. \tag{ST2.6}$$

From the definition the generating function, f has units of inverse fitness and ϕ is unitless. The rescaled equation for the generating function is therefore given by

$$\begin{aligned}\frac{\partial H}{\partial \tau} &= \int d\chi \left[(N\sigma_0)\chi\phi + \frac{\partial_\chi^2 \phi}{2} - \frac{\phi(x)^2}{2} - \chi\partial_\chi \phi + \frac{1}{\sqrt{NU}} \sum_{m=3}^{\infty} \frac{\left\langle \left(s/\langle s^2 \rangle^{\frac{1}{2}} \right)^m \right\rangle \partial_\chi^m \phi}{(m)!(NU)^{(m-3)/2}} \right] \frac{\delta H}{\delta\phi(\chi)} \\ &+ \int d\chi\, d\chi' \left[N\sigma_0 (\partial_\chi \phi)(\chi')^2 - \frac{(\partial_\chi^2 \phi)(\chi')^2}{2} - \frac{\phi(\chi)\phi(\chi')}{2} - (\chi\phi)(\partial_{\chi'}\phi) \right] \frac{\delta H}{\delta\phi(\chi)\delta\phi(\chi')} \\ &- \int d\chi\, d\chi'\, d\chi'' \left[\frac{(\partial_\chi \phi)(\partial_{\chi'}\phi)(\chi'')^2}{2} \right] \frac{\delta H}{\delta\phi(\chi)\delta\phi(\chi')\delta\phi(\chi')} \,.\end{aligned} \tag{ST2.7}$$

Thus, in the limit that $NU \to \infty$ and $Ns \to 0$, the dependence on the higher moments of $\rho(s)$ drops out completely, and we reach the infinitesimal limit where the statistics of the (centered) fitness distribution only depend on the dimensionless product $N\sigma_0 = \sqrt{N^3 U \langle s^2 \rangle}$. An analogous result holds for $G[\phi_0(x), \phi_1(x), t]$, which implies that the dynamics of sequence evolution are subject to this limit as well.

For $0 < N\sigma_0 \ll 1$, we can confirm that this limit is both non-neutral and non-degenerate (i.e., that it still depends on $N\sigma_0$) using the perturbation expansion in [52]. If we had taken $NU \to \infty$ with any other quantity held fixed this would not be the case. For example, we can immediately see from Eq. (ST2.7) that if we had held $\sigma_{\text{det}}^2 = U\langle s \rangle$ constant, then this would yield $N\sigma_0 \to 0$ and hence the neutral limit when $NU \to \infty$ and $Ns \to 0$. On the opposite extreme, if Ns were to fall off more slowly than $1/\sqrt{NU}$, then we would obtain the quasi-degenerate $N\sigma_0 \to \infty$ limit analyzed in Ref. [44].

Calculating the variance in fitness

Although Eq. (ST2.7) is useful for demonstrating the *existence* of the infinitesimal limit, it is far too complicated to solve directly. Fortunately, if we restrict our attention to the average variance in fitness, we can employ a much simpler calculation based on the tunable constraint framework in Ref. [49]. We introduce two new quantities $f(x)$ and $w(x)$, which roughly correspond to the average population density

and the fixation probability at relative fitness x. In the tunable constraint framework, these are related to the population size N and average fitness variance σ^2 through the system of equations

$$\sigma^2\partial_x f(x) = xf(x) - f(x)w(x) + \frac{U\langle s^2\rangle}{2}\partial_x^2 f(x)\,, \tag{ST2.8a}$$

$$-\sigma^2\partial_x w(x) = xw(x) - w(x)^2 + \frac{U\langle s^2\rangle}{2}\partial_x^2 w(x)\,, \tag{ST2.8b}$$

and the normalization conditions

$$1 = \int_{-\infty}^{\infty} f(x)\,dx\,, \qquad \frac{1}{N} = \int_{-\infty}^{\infty} f(x)w(x)\,dx\,. \tag{ST2.8c}$$

A solution to these equations is given in Ref. [49], and we reproduce it here for completeness. We first rescale the relative fitness x by introducing the new coordinate $\chi = x\left(U\langle s^2\rangle\right)^{-1/3}$. Since $w(x)$ and $f(x)$ have the units of fitness and inverse fitness, respectively, we must rescale these as well:

$$\tilde{f}(x) = \left(U\langle s^2\rangle\right)^{1/3} f(x)\,, \quad \tilde{w}(x) = \left(U\langle s^2\rangle\right)^{-1/3} w(x)\,. \tag{ST2.9}$$

In terms of these rescaled variables, our system of equations can be written in the compact form

$$0 = \partial_\chi^2\tilde{f} + 2\alpha\partial_\chi\tilde{f} + 2\chi\tilde{f} - 2\tilde{f}\tilde{w}\,, \tag{ST2.10a}$$

$$0 = \partial_\chi^2\tilde{w} - 2\alpha\partial_\chi\tilde{w} + 2\chi\tilde{w} - 2\tilde{w}^2\,, \tag{ST2.10b}$$

$$1 = \int_{-\infty}^{\infty}\tilde{f}(\chi)\,d\chi\,, \tag{ST2.10c}$$

$$\frac{1}{\beta} = \int_{-\infty}^{\infty}\tilde{f}(\chi)\tilde{w}(\chi)\,d\chi\,, \tag{ST2.10d}$$

where we have introduced the two parameters

$$\alpha = \frac{(N\sigma)^2}{(N\sigma_0)^{4/3}}\,, \quad \beta = (N\sigma_0)^{2/3}\,. \tag{ST2.11}$$

From inspection, we can immediately see that $\tilde{f}(\chi) \propto e^{-2\alpha\chi}\tilde{w}(\chi)$ is a solution to Eq. (ST2.10a), which allows us to eliminate $\tilde{f}$ entirely and yields the simplified system

$$0 = \partial_\chi^2\tilde{w} - 2\alpha\partial_\chi\tilde{w} + 2\chi\tilde{w} - 2\tilde{w}^2\,, \tag{ST2.12a}$$

$$\beta = \frac{\int_{-\infty}^{\infty} e^{-2\alpha\chi}\tilde{w}(\chi)\,d\chi}{\int_{-\infty}^{\infty} e^{-2\alpha\chi}\tilde{w}(\chi)^2\,d\chi}\,, \tag{ST2.12b}$$

where $\tilde{w}(\chi)$ is subject to the boundary conditions $\tilde{w}(\chi) \to 0$ as $\chi \to -\infty$ and $\tilde{w}(\chi) \to \chi$ as $\chi \to \infty$. Thus, a numerical solution of the boundary value problem (for instance, using Matlab's bvp4c function) and a numerical integration of this solution allows us to calculate β as a function of α,

$$\beta = g(\alpha)\,, \tag{ST2.13}$$

and a subsequent inversion yields an expression for $N\sigma$ as a function of $N\sigma_0$,

$$(N\sigma)^2 = (N\sigma_0)^{4/3}g^{-1}[(N\sigma_0)^{2/3}]\,. \tag{ST2.14}$$

Asymptotic formulae can be obtained in the limiting cases that $N\sigma_0 \to \infty$ and $N\sigma_0 \to 0$. Previous work has shown that the $N\sigma_0 \to \infty$ limit requires $\alpha \to \infty$, where the asympotic behavior is given by

$\log g(\alpha) \sim \alpha^3$ [49, 54, 55]. In the opposite limit where $\alpha \to 0$, the differential equation for $\tilde{w}$ reduces to the parameter-free form

$$0 = \partial_\chi^2 \tilde{w} + 2\chi\tilde{w} - 2\tilde{w}^2 \,, \tag{ST2.15}$$

whose solution can be reasonably well-approximated by

$$\tilde{w}(\chi) \approx \begin{cases} \chi & \text{if } \chi > 0, \\ 0 & \text{else.} \end{cases} \tag{ST2.16}$$

The normalization integrals in Eq. (ST2.12) can then therefore be approximated by Γ-functions, and we find that $g(\alpha) \sim \alpha$. Switching notation from α and β back to $N\sigma$ and $N\sigma_0$, these limits imply that

$$N\sigma \sim \begin{cases} N\sigma_0 & \text{as } N\sigma_0 \to 0, \\ \left[4(N\sigma_0)^4 \log(N\sigma_0)\right]^{1/6} & \text{as } N\sigma_0 \to \infty, \end{cases} \tag{ST2.17}$$

which shows that the $N\sigma_0 \to 0$ limit is consistent with neutral coalescent theory.

The expressions in Eqs. (ST2.14) and (S2.17) are valid for *any* distribution of fitness effects $\rho(s)$ as long as the parameters are sufficiently far into the infinitesimal limit. Parameters closer to the boundary of this regime require more careful considerations. For example, if $\rho(s)$ consists of a single deleterious fitness effect, σ^2 must always satisfy

$$\sigma^2 = Us - sR_{\text{ratchet}} \le Us \,, \tag{ST2.18}$$

where $R_{\text{ratchet}} \ge 0$ is the rate of fixation of deleterious alleles due to Muller's ratchet. In order to maintain sensible results throughout the parameter space, we use the modified expression

$$(N\sigma)^2 = \min\left\{(N\sigma_0)^{4/3} g^{-1}\left[(N\sigma_0)^{2/3}\right],\, NU \cdot Ns\right\} \,, \tag{ST2.19}$$

for all of our subsequent calculations. This crude patching does not alleviate all of the inaccuracies near the border of the interference selection regime, but it eliminates any wildly nonsensical behavior. In the $N\sigma \to \infty$ limit, this yields the asymptotic formula

$$N\sigma \sim \begin{cases} \left[2(NU)^2(Ns)^4 \log\left(NU(Ns)^2\right)\right]^{1/6} & \text{if } Ns < NU/2 \log\left(NU(Ns)^2\right), \\ \sqrt{NU \cdot Ns} & \text{else.} \end{cases} \tag{ST2.20}$$

In Figure ST2.1, we compare the predictions in Eq. (ST2.19) with the forward-time simulations in Figure 3. The agreement is generally quite good (certainly better than the naive asymptotics alone), although there are some small systematic disagreements. In particular, we tend to slightly overestimate the variance in fitness at the point where it starts to deviate from the deterministic asymptote $\sigma_{\text{det}}^2 = Us$, and we tend to slightly underestimate it during the transition to the neutral asymptote $\sigma_0^2 = NUs^2$.

Genetic diversity in the large $N\sigma$ limit

In contrast to fitness evolution, there are few analytical tools for predicting genetic diversity in the infinitesimal limit. A notable exception is a recent study by Ref. [44], which explored the genealogical process in the limit that $N\sigma \to \infty$. This limit is easier to analyze than the general case because genetic drift and coalescence are confined to the high-fitness "nose" of the fitness distribution, while the rest of the population can be treated deterministically. Ref. [44] derives asymptotic formulae for the structure of genealogies and the patterns of neutral diversity in this limit.[2] Their results provide valuable qualitative

[2]Similar results were obtained by Ref. [65], although they focused on adapting populations where $NU \ll Ns$.

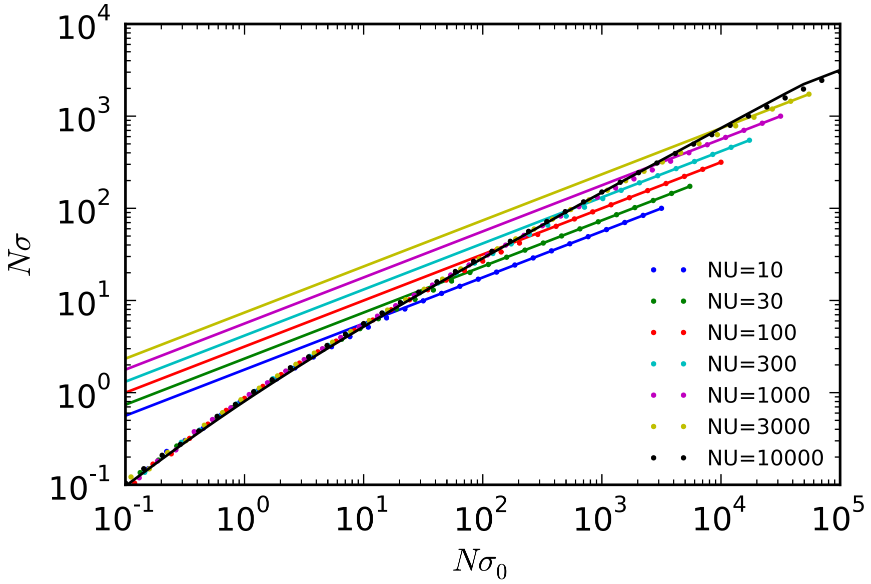

Figure ST2.1. The standard deviation in fitness in our simple purifying selection scenario, as a function of the control parameter $N\sigma_0 = \sqrt{NU(Ns)^2}$. Symbols denote the results of forward-time simulations for the same set of populations included in Figure 3 in the main text. The solid black line denotes the traveling wave prediction from Eq. (ST2.14). For comparison, the colored lines show the deterministic prediction, $\sigma_{\text{det}}^2 = Us$, which applies in the background selection regime.

insights into the evolutionary dynamics of interference, although convergence to this limit is often too slow for quantitative purposes. We briefly review these results here to provide an intuitive contrast with the background selection limit. For the full derivations, see Ref. [44].

As $N\sigma \to \infty$, coalescence predominantly occurs near the nose of the fitness distribution, x_c, which grows as $x_c/\sigma \sim \sqrt{\log(N\sigma)}$ [49, 54, 55]. Thus, in a sample of n individuals most will have relative fitness $x \ll x_c$. Like the background selection limit, this leads to a delay period without coalescence, where the ancestors of the sample migrate towards the nose of the fitness distribution. Since $\sigma/x_c \to 0$, variation in the migration time between individuals can be neglected to leading order, and the delay period has roughly constant duration

$$T_d \;\sim\; \sigma^{-1}\sqrt{6\log\left(N\sigma\right)}\,. \tag{ST2.21}$$

During this time period, all mutations occur as singletons. Once the ancestors of the sample return to the nose, they coalesce uniformly at rate T_c^{-1}, where $T_c \sim T_d$. This is a crucial difference from the background selection limit, where $T_d \ll T_c$. In addition, these coalescence events are drawn from the Bolthausen-Sznitman coalescent [58] rather than the standard Kingman coalescent. The former allows for multiple-merger events, where large fractions of the sample coalesce at the same time. This ancestral process is illustrated in Figure ST2.2, which can be compared with the background selection limit in Figure 1.

Given this genealogical characterization, it is straightforward to derive formulae for linked neutral diversity. The reduction in pairwise heterozygosity scales as

$$\pi/\pi_0 = T_d + T_c \sim (N\sigma)^{-1}\sqrt{24\log\left(N\sigma\right)}\,, \tag{ST2.22}$$

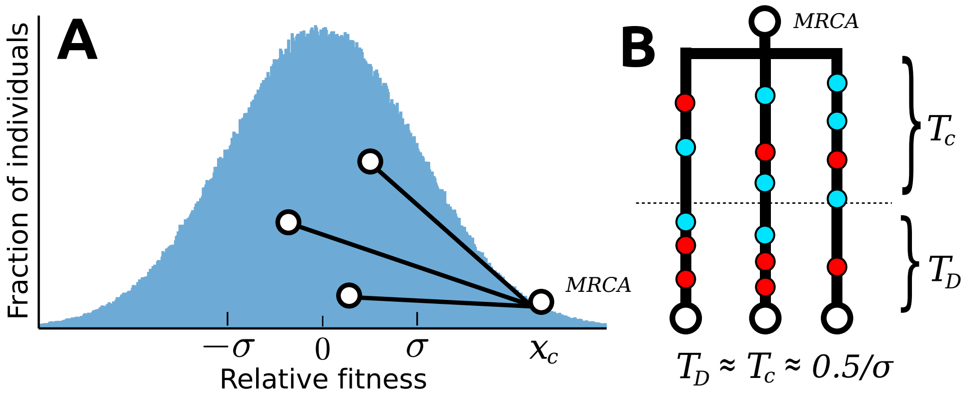

Figure ST2.2. Genealogical structure in the infinitesimal limit when $N\sigma \to \infty$ [44]. Ancestral lineages first experience a delay phase where individuals migrate towards the nose of the fitness distribution. After this point, lineages enter a coalescence phase described by the Bolthausen-Sznitman coalescent [58], which allows for multiple mergers. Selected mutations (red circles) and silent mutations (blue circles) occur at comparable rates in both the delay and coalescence phases.

in constrast to the exponential dependence observed in the background selection limit. When normalized by the total number of segregating sites, the site frequency spectrum is completely independent of $N\sigma$ or any of the other parameters. It is equal to the Bolthausen-Sznitman frequency spectrum but with an additional n units added to the singleton ($i = 1$) class from the initial delay period. As a result, summaries of the site frequency spectrum such as the average minor allele frequency or Tajima's D [31] take on a constant value in the $N\sigma \to \infty$ limit, independent of the underlying parameters. These can be rapidly calculated from coalescent simulations of the Bolthausen-Sznitman coalescent. However, as is apparent in Figure 4, most of the simulated populations in the text have not yet converged to this limiting behavior.
