# Supplementary material for: Genetic Diversity in the Interference Selection Limit
Source: PLoS Genet. 2014 Mar 27;10(3):e1004222. doi: 10.1371/journal.pgen.1004222 (PMC3967937; doi:10.1371/journal.pgen.1004222)
Supplement: Text S3 — The coarse-grained coalescent. (PDF) [file pgen.1004222.s010.pdf]

Text S3 The coarse-grained coalescent

In this section, we provide a more detailed description of the coarse-grained coalescent framework proposed in the main text. For clarity, we will restrict our attention to the asexual limit here, while recombining genomes are treated in Text S4. It will help to recall our original motivation for proposing a new coalescent framework: we want to predict genetic diversity, particularly the distortions in the synonymous site-frequency spectrum, for populations in the interference selection regime where the traditional structured coalescent breaks down.

The most direct approach would be to derive an exact coalescent description of the infinitesimal limit in Text S2, and hope that this description remains valid for finite NU and Ns. Intuitively, this would constitute a "fine-grained" coalescent framework, since we would be approximating a finite number of selected mutations with an infinite continuum of nearly neutral mutations with the same value of $N\sigma$. Initial progress in this direction is given in Refs. [47] and [44], but a complete "fine-grained" coalescent framework does not yet exist.

In the present work, we take the opposite approach. For our simple purifying selection scenario, simulations show that the convergence to the infinitesimal limit is *extremely* rapid — so rapid in fact that we can neglect corrections to this limit all the way up to the boundary of the background selection regime. Thus, it should be possible to use existing structured coalescent predictions along the boundary as a proxy for the infinitesimal limit, and therefore for rest of of the populations the interference selection regime. This intuition suggests the following algorithm for predicting genetic diversity at a particular value of (Ns, NU):

1. Determine whether (Ns, NU) falls in background selection regime or the interference selection regime.

2. In the former case, generate predictions from the structured coalescent evaluated at (Ns, NU) using the algorithm in Text S1.

3. Otherwise, calculate $N\sigma(Ns, NU)$ using Eq. (ST3.19), and find an associated point $(N\tilde{s}, N\tilde{U})$ on the boundary of the interference selection regime where $N\sigma(N\tilde{s}, N\tilde{U}) = N\sigma(Ns, NU)$.

4. Generate predictions from the structured coalescent evaluated at $(N\tilde{s}, N\tilde{U})$.

This algorithm resembles the *patching method* used to approximate the behavior of differential equations [59]. In many respects, it can also be viewed as a *coarse-graining* of the distribution of fitnesses within the population, since $N\tilde{s} > Ns$ and $N\tilde{U} < NU$ by construction. In contrast to the fine-graining method above, where we replace discrete fitness effects by a continuum of infinitesimal mutations, this coarse-graining approach approximates many weakly selected mutations with a smaller number of more strongly selected mutations with the same variance in fitness. However, in order to implement this algorithm, steps (1) and (3) require a precise specification of the boundary between the interference and background selection regimes, which we now describe.

Determining the boundary of the interference selection regime

As is often the case with patching, the precise boundary between the interference and background selection regimes is poorly defined, and there will always be some degree of approximation in fixing a precise location. Analogous behavior arises in the distinction between the "fast" and "slow" regimes of Muller's ratchet, which approximately coincide with the interference selection and background selection regimes discussed in the present work. In the case of the ratchet, the condition $N_e s \sim 1$ is often listed as the boundary between the two regimes, but this is only meant as a rough guide.

Nevertheless, our patching method requires a well-defined boundary. Previous studies have identified several possible candidates, which are based on different features of the background selection limit.

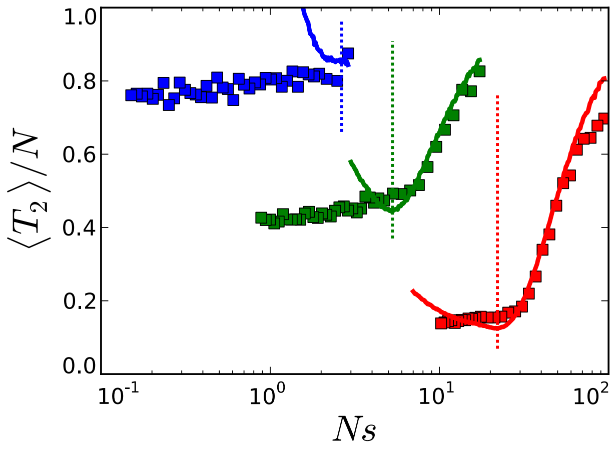

Figure ST3.1. Pairwise heterozygosity along lines of approximately constant fitness variance. Symbols denote the results of forward-time simulations for fixed $N\sigma \approx 2$ (blue), $N\sigma \approx 7$ (green), and $N\sigma \approx 43$ (red), while the solid lines show the predictions from the structured coalescent. The dotted lines denote the corresponding points $(N\tilde{s}, N\tilde{U})$ on the critical line, which are utilized by our coarse-grained theory.

For example, one could examine fluctuations in the fitness class sizes in Eq. (1) [50], the transition in the stochastic dynamics of Muller's ratchet [53], deviations in the fitness variance from the predictions of Eq. (1) (Figure ST2.1), or even the linkage disequilibrium that eventually accumulates among the deleterious mutations (Figure S1). Alternatively, one could use a definition based on the time for ancestral lineages to descend from the least-loaded class compared to their coalescence time within this class [37]. Fortunately, most of these definitions lead to conditions of the form $Nse^{-\lambda} = g(\lambda)$, where $g(\lambda)$ is some polynomial function of λ. It is not clear whether there is any single "correct" definition of the critical line, and the understanding the precise tradeoffs between different possible definitions remains an interesting topic for future work.

In the present study, we have employed a separate definition motivated by the simulation results in Figure ST3.1. We can see that for fixed $N\sigma$, as we tune Ns from $Ns = \infty$ to $Ns = 0$ the structured coalescent predicts a minimum reduction in diversity at a particular value of Ns, below which it rapidly diverges from the simulation results. We propose to use this minimum point as our $N\tilde{s}$, while the corresponding $N\tilde{U}$ can be calculated from the formula $\sigma^2 = \tilde{U}\tilde{s}$. In other words, $N\tilde{s}$ and $N\tilde{U}$ satisfy

$$\left(\frac{\partial T_2^{sc}}{\partial \tilde{s}}\right)_{\sigma^2} = 0\,, \quad \tilde{U} = \frac{\sigma^2}{\tilde{s}}\,, \tag{ST3.1}$$

where T_2^{sc} is the mean pairwise coalescent time predicted by the structured coalescent. According to our coarse-graining algorithm above, the predicted T_2 in the interference selection regime (as a function of $N\sigma$) is therefore given by

$$\frac{T_2}{N}(N\sigma) = \min_{N\tilde{s}} \left\{ \frac{T_2^{sc}}{N} \left(N\tilde{s}, N\tilde{U} = \frac{(N\sigma)^2}{N\tilde{s}} \right) \right\}\,. \tag{ST3.2}$$

For our purposes, we find that T_2^{sc} is well-approximated by the first-order correction given in Eq. (3) in the main text:

$$T_2/Ne^{-\lambda} \sim 1 + \frac{g(\lambda)}{Nse^{-\lambda}}\,; \quad (Nse^{-\lambda} \to \infty)\,, \tag{ST3.3}$$

where

$$g(\lambda) = \int_0^\infty 2\lambda t(1-e^{-t})e^{-t-2\lambda e^{-t}+\lambda e^{-2t}}\,dt\,. \tag{ST3.4}$$

For small λ, we have $g(\lambda) \sim 3\lambda/2$, while for large λ we have $g(\lambda) \sim \log 2\lambda$. We can use this formula to obtain an analytical version of the condition for $N\tilde{s}$ and $N\tilde{U}$ in Eq. (ST3.1). Differentiating Eq. (ST3.3), we find that

$$N\tilde{s} = \int_0^\infty t(1-e^{-t})e^{-t+\left(\frac{\sigma}{\tilde{s}}\right)^2(1-e^{-t})^2}\left[1+(4-2e^{-t})\left(1-\left(\frac{\sigma}{\tilde{s}}\right)^2 e^{-t}\right)\right]dt\,, \tag{ST3.5a}$$

$$N\tilde{U} = (N\sigma)^2/N\tilde{s}\,. \tag{ST3.5b}$$

In the limit that $N\sigma \to \infty$, we can invert these equations to obtain $N\tilde{s} \sim N\sigma\log^{-1/2}(N\sigma)$ and $N\tilde{U} \sim N\sigma\log^{1/2}(N\sigma)$. Thus, our expression for T_2 in the interference selection regime is (up to logarithmic corrections) given by

$$\frac{T_2}{N} \sim \frac{1}{N\sigma}\,; \quad (N\sigma \to \infty)\,, \tag{ST3.6}$$

which agrees with the "fine-grained" asymptotic scaling in Eq. (ST3.22). For more accurate predictions, we must invert Eq. (ST3.5) numerically to obtain $(N\tilde{s}, N\tilde{U})$ as a function of $N\sigma$ (see Methods).

Accuracy of the coarse-grained predictions

We now examine the accuracy of our coarse-grained predictions in slightly more detail. First, we recall that in the main text we focused on ensemble averages of various diversity statistics. It is therefore natural to ask whether the coarse-grained predictions hold for *distributional properties* as well. One advantage of our formal analysis in Text S2 is that it shows that higher-order moments also become invariant in the infinitesimal limit. Thus, the accuracy of our coarse-grained predictions depends primarily on how quickly this limit is approached, and whether the structured coalescent remains accurate near the border of the background selection regime.

In Figure ST3.2, we plot the distribution of synonymous heterozygosity for two different populations in the interference selection regime, as well as the corresponding distributions generated by structured coalescent simulations of the coarse-grained parameters. Under the independent sites assumption, this should yield a geometric distribution with mean $2N_eU_n$. Instead, we observe an increasingly strong peak in π with increasing $N\sigma$, and a corresponding reduction in the variance relative to the neutral expectation [36, 37]. The effective population size approximation completely neglects this qualitative transition, while the coarse-grained predictions are both qualitatively and quantitatively quite accurate.

We can quantify the peaked nature of this distribution over a broader range of parameters by focusing on the variance in π and constructing a collapse plot analogous to Figure 4 in the main text. Again, under the effective population size approximation, the variance is equal to its neutral expectation,

$$\frac{\mathrm{Var}(\pi)}{E(\pi)^2} = 1 - \frac{1}{E(\pi)} \approx 1\,; \quad (N\sigma \to 0)\,, \tag{ST3.7}$$

which is independent of N_e or any of the other parameters. On the other hand, when $N\sigma \to \infty$ the distribution of T_2 approaches a convolution of an exponential distribution and a constant delay with the same mean, which implies that

$$\frac{\mathrm{Var}(\pi)}{E(\pi)^2} \approx \frac{1}{2}\,; \quad (N\sigma \to \infty)\,, \tag{ST3.8}$$

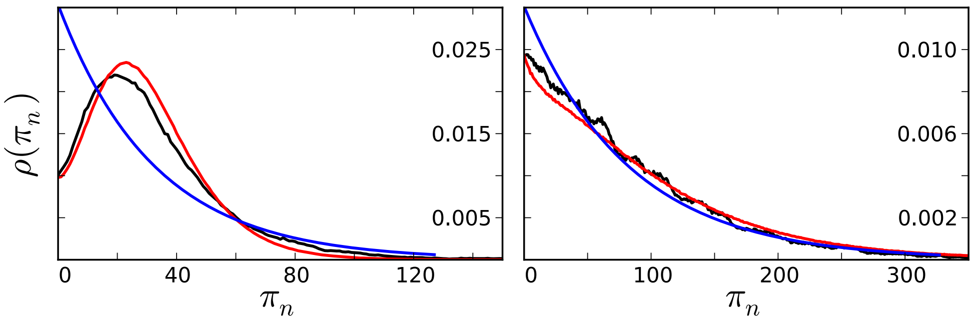

Figure ST3.2. Distribution of pairwise diversity in the interference selection regime. The distribution of neutral pairwise heterozygosity for a population with $Ns = 5$ (left) and $Ns = 0.25$ (right), with $NU = NU_n = 50$. Black lines denote the results of forward-time simulations and the red lines show the structured coalescent predictions for our coarse-grained theory. For comparison, the blue lines show the single-locus predictions with N_e fitted from the mean of the forward-time distribution.

In Figure ST3.3, we measure the variance for each of the populations in Figure 4 from the main text, and we compare these with our coarse-grained predictions. Some inaccuracies are apparent, particularly for large $N\sigma$ where the coarse-grained predictions overshoot the $N\sigma \to \infty$ limit in Eq. (ST3.8). However, our predictions still capture much of the qualitative dependence that is completely neglected by the effective population size approximation.

The errors observed in Figure ST3.3 can also be seen in several of the other figures in the main text. This is not too surprising, since our coarse-grained model is merely an approximation of the coalescent process in the interference selection regime. Deviations from our coarse-grained predictions can arise for three distinct reasons:

1. Convergence to the infinitesimal limit is too slow, and the equivalence between strongly-interfering populations with the same $N\sigma$ is inaccurate.

2. Our expression for $N\sigma$ in Eq. (ST3.19) is inaccurate, and populations are not matched with the proper coarse-grained parameters.

3. The structured coalescent is inaccurate for the coarse-grained parameters $(N\tilde{s}, N\tilde{U})$.

A nice feature of the collapse plots in Figure ST3.3 (and the main text) is that they can be used to distinguish these different sources of error. For example, it is clear from Figure ST3.3 and Figure 4 in the main text that errors of type (1) are present. Populations closer to the infinitesimal limit are slightly "less neutral" than their counterparts near the boundary with the same $N\sigma$. However, these deviations are relatively small and contribute only a small portion of the error in the coarse-grained predictions. In a similar vein, we can see from Figure ST2.1 that errors of type (2) are present, particularly near the boundary between the infinitesimal limit and the deterministic formula $\sigma^2_{\text{det}} = Us$. Yet these errors are also rather small.

In contrast, we see from Figure 4 and Figure ST3.3 that the largest errors in the coarse-grained model stem from inaccuracies in the structured coalescent near the boundary of the interference selection regime. Coarse-grained parameters with $N\sigma \sim O(1)$ are consistently predicted to be more neutral than is observed in simulations, while those populations with extremely large $N\sigma$ are predicted to be less neutral than is observed. Errors such as this are a natural consequence of our crude patching method, which approximates the $Ns \to 0$ limit with corrections designed for the $Ns \to \infty$ limit. In addition, as we saw in Text S1, the structured coalescent corrections are asymptotically incorrect even in the limit

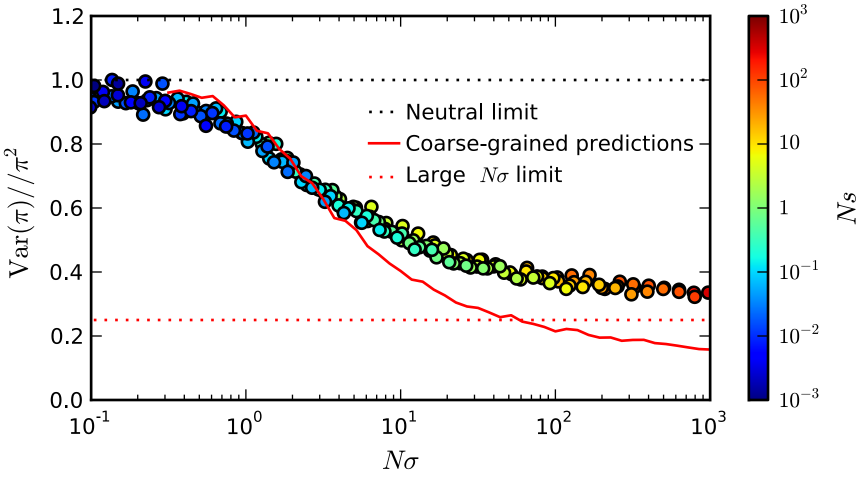

Figure ST3.3. The relative variance in pairwise (synonymous) diversity as a function of $N\sigma$ for the same populations as Figure 3 in the main text. Again, symbols denote the results of forward-time simulations, while the solid red line gives the structured coalescent predictions of our coarse-grained theory. For comparison, we have also included predictions from the neutral limit, as well as the $N\sigma \to \infty$ limit from Ref. [44].

that $Ns \to \infty$, since they do not include the effects of fitness class fluctuations that formally enter at the same order as the corrections in Eq. (ST3.3). In this sense, it is surprising that the structured coalescent predictions are accurate at all, even well into the background selection regime.
