# Supplementary material for: Genetic Diversity in the Interference Selection Limit
Source: PLoS Genet. 2014 Mar 27;10(3):e1004222. doi: 10.1371/journal.pgen.1004222 (PMC3967937; doi:10.1371/journal.pgen.1004222)
Supplement: Text S4 — Recombining genomes. (PDF) [file pgen.1004222.s011.pdf]

Text S4 Recombining genomes

In contrast to the asexual populations in the previous sections, relatively few methods exist for predicting the evolution of recombining genomes. The primary difficulty stems from the fact that recombination requires explicit haplotype information: the fitness of a recombinant offspring depends not only on the fitness of each parent, but also on the precise location of the mutations within each parental genome. As a result, we no longer have a clean separation of scales between the *mesoscopic* dynamics of fitness evolution and the *microscopic* dynamics of sequence evolution that proved so useful in the analysis of asexual populations. Rather, both effects must be modeled simultaneously. For similar reasons, forward-time simulations of recombining genomes are significantly more time-consuming than their asexual counterparts, even when we are only interested in evolution at the fitness level.

Recombination in the background selection regime

Because of these difficulties, most earlier work on recombining genomes falls back on the independent-sites assumption described in the main text. This avoids the haplotype problem by assuming that (on evolutionary timescales) the frequencies of individual mutations are in linkage equilibrium with each other, where they evolve with some effective population size N_e. In the simple model studied here, the effective population size can be calculated in the background selection limit, which yields the well-known formula $N_e = Ne^{-2U/(2s+R)}$ from Eq. (6). This prediction is valid in the limit that $Nse^{-2U/(2s+R)} \to \infty$ with NU/Ns and NR/Ns fixed, which is similar to what we found in the asexual case. In this limit, the linkage disequilibrium between two sites separated by a fixed map length ΔR scales as

$$LD \sim 1/(N_e \Delta R)\,, \tag{ST4.1}$$

which self-consistently vanishes in agreement with the independent-sites assumption above.

However, for finite $N_e s$ it is known that selection causes distortions in neutral allele frequencies similar those observed in asexual populations, although the dependence on the underlying parameters is somewhat different. A structured coalescent description has only recently been derived in this limit [42, 61], and its analytical implications are still being explored [38]. A cursory reading of Ref. [61] could give the impression that the recombining structured coalescent avoids the interference issues that plagued its asexual counterpart, but we have shown in Figure S1 that this is not the case. We see that while the structured coalescent captures much of the distortion when $Nse^{-2U/(2s+R)} \gg 1$, it also rapidly diverges from simulation results near its maximum predicted deviation from neutrality, similar to the asexual case above. This leaves a broad "interference selection regime" in recombining populations as well, even when the ratio of mutation and recombination rates is not too large.

Interference and the linkage block ansatz

In order to predict the diversity in this interference selection regime, the most direct approach would be to the extend the coarse-graining in Text S3 to the recombining structured coalescent. However, this direct approach is more difficult than it appears because of the tight coupling between genetic diversity and fitness evolution in recombining genomes. Even if the interference selection *limit* still exists in recombining genomes (for fixed NR), we can no longer predict the variance in fitness within these populations without first characterizing the deleterious diversity along the chromosome. In asexual populations, this calculation was crucial for connecting the interference selection regime to the proper coarse-grained model. Moreover, even if the direct approach was successful, the recombining structured coalescent is sufficiently complicated that it would provide little insight into the influence of recombination rate in these populations. This is arguably the most important goal of any theoretical analysis.

For these reasons, we eschew the direct approach here in favor of a simple heuristic argument, which trades some mathematical rigor for enhanced qualitative insight — and ultimately, better quantitative

predictions. Like the ordinary independent sites assumption, our heuristic approach is based on the fact that distant parts of the genome are effectively independent of each other. Yet this intuition cannot apply all the way down to the single-site level. Rather, evolution on sufficiently short length scales will resemble an asexual genome, where interference builds up more rapidly than recombination can act to remove it. To the extent that this transition is sharp, the evolution of a recombining genome can be viewed as a set of freely recombining *linkage blocks*, within which evolution is effectively asexual.

We argued in the text that the length of these blocks, L_b/L, must scale as

$$\frac{L_b}{L} \sim \begin{cases} c/T_2R & \text{for } T_2R \to \infty, \\ 1 & \text{for } T_2R \to 0, \end{cases} \tag{ST4.2}$$

where c is some $\mathcal{O}(1)$ constant. The motivation for this scaling is simply that (up to logarithmic corrections) T_2 is relevant timescale over which genetic and phenotypic diversity is accumulated, and that blocks of size L_b experience $\sim \mathcal{O}(1)$ recombination events over this time period. Previous work has also shown that L_b/L corresponds to the extent of linkage disequilibrium in the background selection regime [42]. For concreteness, we choose the functional form

$$\frac{L_b}{L} = \left(1 + \frac{T_2R}{4}\right)^{-1}, \tag{ST4.3}$$

which satisfies the scaling in Eq. (ST4.2) and seems to yield good results in practice. Given this definition, we partition the genome into asexual blocks of size L_b which evolve independently of each other, and whose behavior can be predicted with the asexual methods described above. In the interference selection regime, this implies that:

1. The coalescent timescale T_2 is set not by the *total* variance in fitness within the population, but rather by the fraction $\sigma^2 \cdot (L_b/L)$ that accumulates within a single linkage block.

2. The functional relationship between T_2 and $\sigma^2 \cdot (L_b/L)$ is given by the asexual formula Eq. (ST4.2) derived in Text S3.

3. The fractional variance $\sigma^2 \cdot (L_b/L)$ can be predicted from the asexual formula in Eq. (ST4.19), but with an *effective mutation rate* $U_{\text{eff}} = U \cdot (L_b/L)$.

We verify these predictions in Figures ST4.1 and ST4.2 using the same forward-time simulations from Figure 4 in the main text. We see that our simple approximation is surprisingly accurate: $U \cdot (L_b/L)$ determines $\sigma^2 \cdot (L_b/L)$, and $\sigma^2 \cdot (L_b/L)$ in turn determines T_2. Since L_b/L is itself a function of T_2, we obtain a closed system of equations and a self consistency condition for the linkage scale:

$$\frac{L_b}{L} = \frac{1}{1 + \frac{NR}{4} \cdot \frac{T_2}{N}\left(N\sigma\left(NU \cdot \left(\frac{L_b}{L}\right), Ns\right)\right)}, \tag{ST4.4}$$

where T_2/N is given by Eq. (ST4.2) and $N\sigma(NU, Ns)$ is given by Eq. (ST4.19). In the limit that $T_2R \gg 1$ and $\sigma^2 \ll Us$, this simplifies to

$$\left(\frac{1}{4}\right) \cdot (N\sigma_{0,\text{eff}})^2 \cdot \frac{T_2}{N}\left(N\sigma(N\sigma_{0,\text{eff}})\right) = \left(\frac{U}{R}\right) \cdot (Ns)^2, \tag{ST4.5}$$

where $N\sigma_{0,\text{eff}} = \sqrt{NU(Ns)^2 (L_b/L)}$ is the effective control parameter for the interference selection regime on the linkage block. This implies that any two populations with the same value of $U/R \cdot \langle (Ns)^2 \rangle$ should possess the same patterns of synonymous diversity, on average. This quantity has a natural interpretation as the fitness variance within the typical LD scale that would be obtained if fitness was not a selected

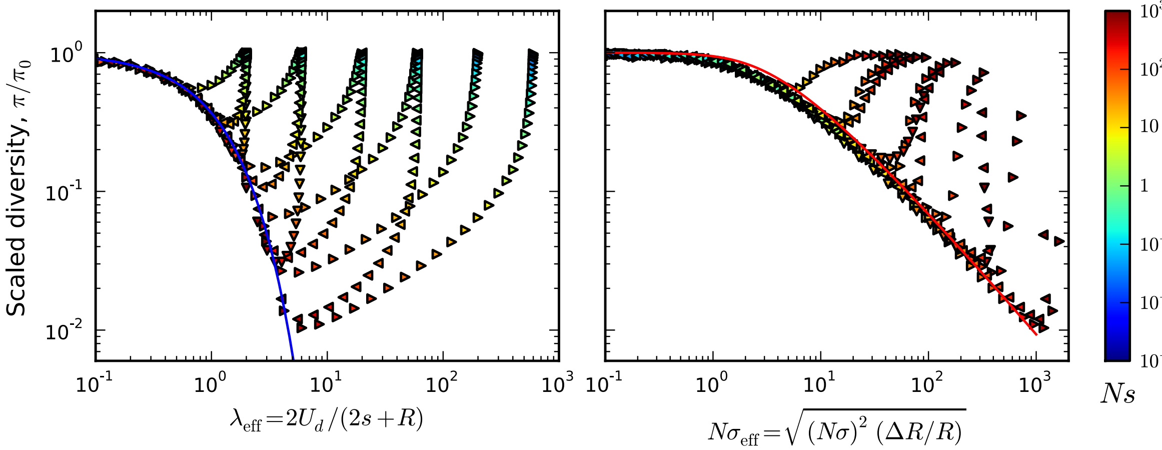

Figure ST4.1. The reduction in synonymous diversity in the presence of recombination. Colored points are measured from forward-time simulations of our simple purifying selection scenario for $NR = 10$ (right triangles) and $NR = 100$ (left triangles). Other parameters are $Ns \in (10^{-3}, 10^3)$ and $NU = 10$, 30, 100, 300, 10^3, 3×10^3. In the left panel, these results are plotted as a function of the background selection parameter $\lambda_{\rm eff} = 2U/(2s + R)$, and the prediction from Eq. (6) is given by the dashed line. The right panel shows the same set of results plotted as a function of the variance in fitness per linkage block, where both $N\sigma$ and $\Delta R/R = (1 + T_2R/4)^{-1}$ are measured from the simulations. The solid red line gives the (asexual) prediction from Eq. (ST4.2).

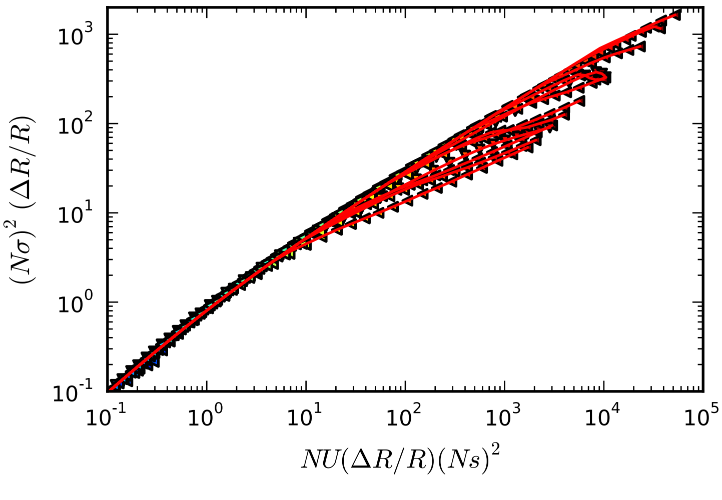

Figure ST4.2. The variance in fitness in recombining populations, as a function of the effective mutation rate $U_{\rm eff} = U(\Delta R/R)$. Symbols denote the same set of forward-time simulations as Figure ST4.1, where $N\sigma$ and $\Delta R/R$ are again measured from the simulations. The red lines show the (asexual) predictions from Eq. (ST4.19). Deviations from the master curve denote the transition to the deterministic limit $\sigma^2_{\rm det} = Us$.

trait. When $N\sigma_{0,\text{eff}} \gg 1$, we can employ the asymptotic formulae in Eqs. (ST4.17) and (S4.6) to simplify these expressions even further. Up to logarithmic corrections, we find that

$$\frac{L_b}{L} \sim \sqrt{\frac{U\langle s^2\rangle}{R^3}}\,, \tag{ST4.6a}$$

$$T_2 \sim \sqrt{R/(U\langle s^2\rangle)}\,, \tag{ST4.6b}$$

$$\sigma^2 \sim \sqrt{RU\langle s^2\rangle}\,, \tag{ST4.6c}$$

which are only weakly dependent on N. This gives some intuition for the scaling behavior, but many biologically relevant parameters lie outside this asymptotic regime and therefore require a numerical solution of Eq. (ST4.4) to calculate L_b/L (see Methods). Once L_b/L is determined, we can generate predictions for the site frequency spectrum by applying our *asexual* coarse-grained model for the parameters $NU \cdot (L_b/L)$ and Ns. As a side benefit, our calculation of σ^2 gives a novel prediction for the rate of Muller's ratchet in sexual populations,

$$R_{\text{ratchet}} = \frac{\sigma^2}{s} - U\,, \tag{ST4.7}$$

which has important implications for the evolution of sex and genome architecture [87].

While the accuracy of the linkage block approximation is encouraging, some small systematic errors remain. These are already apparent from Figure 4, where each value of NR appears to collapse to a *slightly* different curve, despite the fact that the collapse within each value of NR is quite good. These errors are likely caused by a crucial factor we neglected in our original analysis: distant regions of the genome may be *independent*, but they still influence each other's evolution through a reduction in the effective population size [46, 67, 71, 72]. Fitness variation at a distant locus represents effectively non-heritable variance in offspring number when the time T_r between successive recombination events satisfies $T_r \ll T_{MRCA}$, which would occur if the loci were located on different linkage blocks. Existing studies have focused on these effects at the level of individual sites, but extending this intuition to the linkage blocks, we might expect corrections to N_e which depend on products of the form

$$\sigma^2\left(\frac{L_b}{L}\right)\left[n \cdot R \cdot \left(\frac{L_b}{L}\right)\right]^{-2}\,, \tag{ST4.8}$$

where n represents the distance measured in the number of linkage blocks. The power-law decay with n suggests that even relatively distant blocks contribute to the reduction in N_e, similar to the background selection limit. This is consistent with the qualitative observation that longer genomes (i.e., larger values of NR) have a shallower "distortion vs diversity curve" in Figure 4, since they have more linkage blocks to contribute to the reduction in N_e, and therefore, the reduction in π/π_0. Unfortunately, quantitative predictions of N_e are difficult, since the transition between the effectively asexual and effectively unlinked regimes is not sufficiently sharp to apply existing theory. A more detailed analysis of distant linkage blocks remains an important avenue for future work.
